# Predictability in process-based ensemble forecast of influenza

**DOI:** 10.1371/journal.pcbi.1006783

**Published:** 2019-02-28

**Authors:** Sen Pei, Mark A. Cane, Jeffrey Shaman

**Affiliations:** 1 Department of Environmental Health Sciences, Mailman School of Public Health, Columbia University, New York, NY, United States of America; 2 Lamont-Doherty Earth Observatory, Columbia University, New York, NY, United States of America; Yale School of Public Health, UNITED STATES

## Abstract

Process-based models have been used to simulate and forecast a number of nonlinear dynamical systems, including influenza and other infectious diseases. In this work, we evaluate the effects of model initial condition error and stochastic fluctuation on forecast accuracy in a compartmental model of influenza transmission. These two types of errors are found to have qualitatively similar growth patterns during model integration, indicating that dynamic error growth, regardless of source, is a dominant component of forecast inaccuracy. We therefore examine the nonlinear growth of model initial error and compute the fastest growing directions using singular vector analysis. Using this information, we generate perturbations in an ensemble forecast system of influenza to obtain more optimal ensemble spread. In retrospective forecasts of historical outbreaks for 95 US cities from 2003 to 2014, this approach improves short-term forecast of incidence over the next one to four weeks.

## Author summary

Mathematical models are now used to forecast infectious disease incidence at the population scale. By better understanding how errors in prediction systems are introduced, grow and impact the predictability of infectious disease, forecast accuracy could be improved. Here we explore the growth pattern of errors introduced from two major sources–model initial conditions and stochastic fluctuation–in a simple, compartmental model describing influenza transmission. We find that model initial error typically undergoes faster growth due to nonlinear amplification during model evolution. Adopting techniques used in numerical weather prediction, we leverage this growth of uncertainty and modify an ensemble forecast system to generate optimal perturbations along the fastest growing direction of initial error. This perturbation procedure increases ensemble spread, which better captures observations with large uncertainties. In retrospective forecasts for 95 US cities during the 2003 through 2014 flu seasons, this procedure leads to a substantial improvement of short-term forecast quality.

## Introduction

Influenza imposes a tremendous toll on global public health due to its recurrent worldwide spread and associated heavy morbidity and mortality burden [1]. To better prepare for and mitigate future outbreaks, accurate forecasts of influenza transmission are needed. Over the last few years, a number of forecasting systems have been developed and operationalized in the hopes of informing real-time policy-making during an influenza outbreak [2–11]. Although forecast skill has advanced significantly, the predictability of nonlinear influenza transmission dynamics is limited by the errors in model forecast systems [12]. These errors derive from three major sources: errors in model initial conditions, stochasticity in model dynamics, and model misspecification. To further improve influenza forecast accuracy, a better understanding of these errors and their impact on forecast uncertainty is needed. In this work, we focus on the first two error sources (i.e., initial condition error and stochasticity) and do not investigate model misspecification.

While prediction uncertainty and error growth in weather and climate forecasting has been well studied [13–23], few works have examined this phenomenon in forecast models of infectious disease. In this work, we perform an analysis of prediction uncertainty and error growth in a compartmental model of influenza transmission. We compare growth patterns of errors derived from both initial condition error and stochastic fluctuation during different stages of an influenza outbreak. We find these error sources have similar effects on influenza incidence predictability; however, initial error leads to a faster increase in ensemble spread and therefore appears more responsible for the degradation of predictability. We then derive the linear propagator of the transmission model and calculate the unstable direction of initial error growth using singular vector analysis [14–17]. The flow-dependent singular vectors obtained can then be used to generate optimal perturbations during the ensemble forecast of influenza, an adaptation of methods used in operational numerical weather prediction [21–23]. We optimize this perturbation procedure in a model-data assimilation forecast framework and validate it using historical outbreaks from 95 cities in the United States from 2003 to 2014. Compared with the baseline method without optimal perturbations, the properly perturbed system substantially improves short-term forecast quality around and after the peak of an outbreak, when observed incidence levels are most uncertain. This procedure of diagnosing and optimally perturbing ensemble forecasts of influenza can be applied to ensemble forecast systems for other infectious diseases.

## Materials and methods

### Data

We combine Google Flu Trends (GFT) data and concurrent laboratory-confirmed influenza positivity rates to generate observational estimates of influenza incidence. Using internet search query activity, GFT provided real-time estimates of weekly influenza-like illness (ILI) per 100,000 people seeking medical treatment for major cities in the United States during 2003–2015 [24]. ILI is a medical diagnosis of possible influenza or other illness defined by symptoms of a fever above 37.8 °C plus cough and/or sore throat. These symptoms are not exclusively caused by influenza, as other respiratory viruses, e.g., respiratory syncytial virus, rhinovirus, may produce similar symptoms. Therefore, to capture a more specific signal of influenza infection incidence, we multiply weekly municipal GFT ILI with the percentage of laboratory-confirmed influenza infections among patients presenting with ILI, compiled regionally through the National Respiratory and Enteric Virus Surveillance System and US-based World Health Organization collaborating laboratories [25]. This combined metric, termed ILI+, better tracks influenza incidence and thus provides a more specific target for inference and forecast [5,25]. Excluding the pandemic seasons of 2008–2009 and 2009–2010, locations without absolute humidity data, and seasons with incomplete observations, we used 790 ILI+ outbreak time series from 95 cities in the US during the 2003–2004 through 2013–2014 seasons in this study.

### Humidity-driven SIRS model

A parsimonious SIRS (susceptible-infected-recovered-susceptible) model forced by absolute humidity (AH) conditions is used to simulate influenza activity. This SIRS model with environmental forcing, previously validated against historical outbreaks in the United States [26,27], provides a concise mathematical description of influenza transmission dynamics. Within an assumed uniformly mixed population, transmission proceeds according to the following equations:

$$\frac{dS}{dt}=\frac{N-S-I}{L}-\frac{\beta \left(t\right)IS}{N},$$ (1)

$$\frac{dI}{dt}=\frac{\beta (t)IS}{N}-\frac{I}{D},$$ (2)

where *N*, *S* and *I* are the total, susceptible and infected populations, respectively; *β*(*t*) is the contact rate at time *t*; *D* is the mean infectious period; and *L* is the average duration of immunity. As population size is constant, the recovered population is *N* − *S* − *I*. The contact rate *β*(*t*) is modulated by local AH conditions via

$$R_{0}(t)=\beta (t)D=e^{a\times q(t)+b}+R_{0min}.$$ (3)

Here, *R*_0_(*t*) is the basic reproductive number (the expected number of secondary infections generated by a single infection in a fully susceptible population), and *q*(*t*) is specific humidity (a measure of AH). The coefficients in the exponential term are estimated from laboratory experiments on influenza virus survival: *a* = −180 and *b* = *log*(*R*_0*max*_ − *R*_0*min*_), where *R*_0*max*_ and *R*_0*min*_ are the maximum and minimum basic reproductive numbers [27]. Local AH conditions, i.e., daily climatological humidity data averaged from 1979 through 2002, are derived from the North American Land Data Assimilation System [28].

The SIRS model can be integrated forward in time either deterministically or stochastically. When inspecting the growth of initial error, the model was run deterministically using a fourth-order Runge-Kutta stepping scheme. A stochastic version was used to examine the impact of stochastic fluctuation. There exist a number of approaches for introducing stochasticity into model dynamics [29–35]. Here we used an event-driven approach that interprets the transitions between individuals’ states as Markov chains [31]. In particular, the rate for each type of transition event, defined in Eqs 1 and 2 (e.g., susceptible to infected, infected to recovered, and recovered to susceptible), in a short time step *δt* = 1 was perturbed through multiplication with a Gamma distributed parameter $\gamma ∼G(1/\sigma_{p}^{2},\sigma_{p}^{2})$ (mean 1 and standard deviation *σ*_*p*_). Mathematically, the model equations are modified to

$$\frac{dS}{dt}=\frac{N-S-I}{L}\gamma_{R\to S}-\frac{\beta (t)IS}{N}\gamma_{S\to I},$$ (4)

$$\frac{dI}{dt}=\frac{\beta (t)IS}{N}\gamma_{S\to I}-\frac{I}{D}\gamma_{I\to R},$$ (5)

where *γ*_*S*→*I*_, *γ*_*I*→*R*_ and *γ*_*R*→*S*_ represent the stochastic forcing on the transition events from susceptible to infected, infected to recovered, and recovered to susceptible, respectively. The exact number of individuals transitioning from one state to another during a time step *δt* = 1 was generated from a Poisson distribution with the mean value set equal to the value in the deterministic process. This approach has been widely used to model the stochastic dynamics of infectious disease [30–35].

In all model simulations, the total population was set as *N* = 1 × 10^5^ uniformly. Because ILI+ (i.e., influenza infection per 100,000 patient visits) is reported as a rate not a magnitude, the total population size, *N*, is arbitrary. To generate synthetic outbreaks, initial conditions (*S*,*I*) and epidemiological parameters (*R*_0*max*_, *R*_0*min*_, *D*,*L*) were drawn randomly using a Latin hypercube sampling strategy [36] from the following ranges: 3,000 ≤ *S* ≤ 8,000, 0 ≤ *I* ≤ 1,000, 1.3 ≤ *R*_0*max*_ ≤ 4, 0.8 ≤ *R*_0*min*_ ≤ 1.3, 2 days ≤ *D* ≤ 7 days, 1 year ≤ *L* ≤ 10 years. [In the two-dimensional case, Latin hypercube sampling generates *n* samples in two steps: 1) divide the state-space into *n* × *n* uniform squares and 2) select sample positions such that there is only one sample in each row and each column. High-dimensional Latin hypercube sampling is a generalization of this process.] The humidity-driven SIRS model was integrated from October 1^st^ for 40 consecutive weeks to generate synthetic outbreaks. Weekly observations of local influenza incidence are the number of new infections, *O*_*t*_, which are calculated during model integration. To mimic real-world observational error, random Gaussian noise with mean 0 and observation error variance $OEV_{t}=5\times 10^{3}+{\left(\sum_{j=t-3}^{t-1}O_{j}/3\right)}^{2}/50$ at week *t* was added to the simulated weekly incidence.

### The ensemble adjustment kalman filter

Data assimilation methods were used to infer unobserved variables and parameters in the humidity-driven SIRS model from observations. Specifically, we employed a sequential ensemble filtering algorithm called the Ensemble Adjustment Kalman Filter (EAKF) [37] to iteratively optimize the distribution of variables (*S*,*I*) and parameters (*R*_0*max*_, *R*_0*min*_, *D*,*L*) with each successive observation. While the EAKF is optimal for linear systems, it also exhibits satisfactory performance in practice for weakly nonlinear dynamical models such as the SIRS model we study here. To date, the EAKF has been used for the inference and forecast of a number of infectious diseases, such as influenza [4–6,38–40], West Nile Virus [41–42], dengue [43], respiratory syncytial virus [44], Ebola [45] and antibiotic-resistant pathogens [46].

To represent the state-space distribution, the EAKF maintains an ensemble of system state vectors acting as samples from the distribution. The EAKF assumes that both the prior distribution and likelihood are Gaussian and can be fully characterized by the first two moments, i.e., mean and covariance. Unobserved variables and parameters are updated through their covariability with the observed state variable, which can be computed directly from the ensemble. In the EAKF, the variables and parameters are updated deterministically so that higher moments of the prior distribution are preserved in the posterior [37].

The SIRS model-EAKF system can simulate the behavior of realistic epidemic curves due to the iterative adjustment of the system state by the EAKF. In S1 Text, we fit historical outbreaks from New York, Denver, Los Angeles and Houston for the 2010–2011 to 2013–2014 seasons. In general, the posterior estimate captures the ILI+ curves in these outbreaks (see S1 Text, Fig A).

## Results

### Analytical and numerical investigation of error growth

#### Roles of model initial error and stochastic forcing

The predictability of a dynamical system can be measured by the variance of an ensemble of perturbed trajectories [13]. For *n* model trajectories perturbed at time *t*, we denote *f*_*i*_(*t*,*k*) (*i* = 1,⋯,*n*) as the observation of the *i*th trajectory after time *k*. The ensemble spread is defined as

$$\sigma^{2}(t,k)\equiv \frac{1}{n}\sum_{i=1}^{n}{\left[f_{i}(t,k)-\bar{f}(t,k)\right]}^{2},$$ (6)

where $\bar{f}(t,k)$ is the ensemble mean over all trajectories, i.e., the mean of *f*_*i*_(*t*,*k*) (*i* = 1,⋯,*n*).

The humidity-driven SIRS model was perturbed in two different ways. For the first, at time *t* we perturbed the initial condition of variables (*S*_*t*_,*I*_*t*_) through multiplication with scaling parameters (*ε*_1_,*ε*_2_), where both *ε*_1_ and *ε*_2_ were generated from a Gaussian distribution $N(1,\sigma_{p}^{2}).$ For each synthetic outbreak and each day of perturbation, we generated *n* = 100 perturbed trajectories and tracked the evolution of the ensemble spread for time *k*. For the second, at each perturbation time *t*, we simulated *n* = 100 realizations of the stochastic model (Eqs 3 and 4) using a Gamma distribution $G(1/\sigma_{p}^{2},\sigma_{p}^{2})$ with the same variance $\sigma_{p}^{2}$, starting from the same initial condition (*S*_*t*_,*I*_*t*_). Note that the first perturbation method produces errors in initial conditions and integrates the model deterministically; the second perturbation method integrates the model from the same initial condition but introduces errors through continuous stochastic forcing of model dynamics. Because the above two perturbation methods operate in different ways, it is challenging to design a completely controlled, fair comparison. Here, we impose perturbations with the same variance $\sigma_{p}^{2}$ in order to control the strength of the initial condition perturbation and the intensity of stochastic forcing.

**Fig 1:**
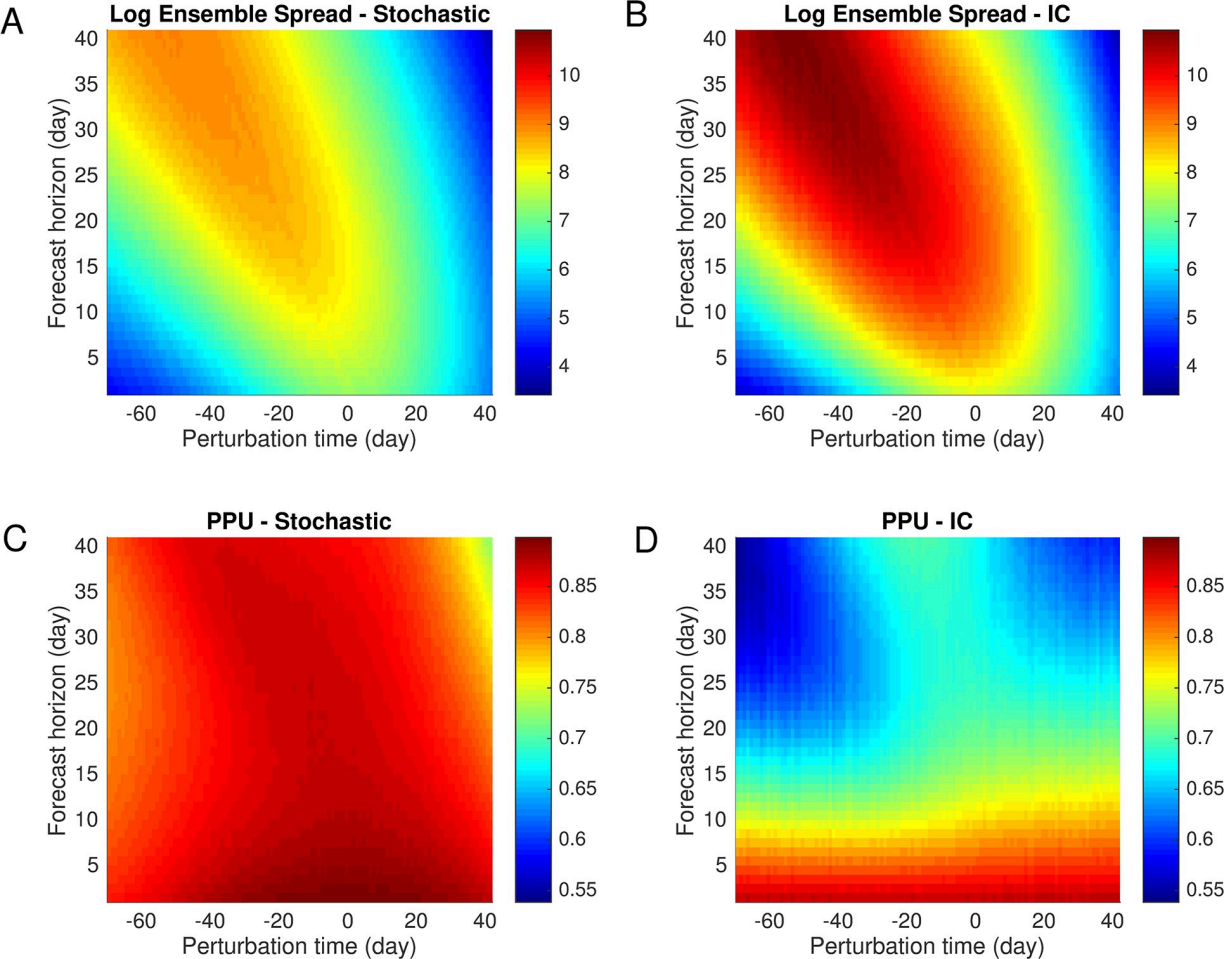
Log-transformed ensemble spread and potential prediction utility (PPU) for stochastic forcing and initial error. We generated 1,000 synthetic outbreaks forced by daily absolute humidity for New York City. Perturbations were imposed at a given time relative to the outbreak peak (-70 days to 42 days); the evolution of log-transformed ensemble spread (base *e*) for the following 6 weeks (42 days) is displayed. A negative/positive perturbation time indicates the model is perturbed before/after the peak. Perturbations were generated from a gamma distribution $G(1/\sigma_{p}^{2},\sigma_{p}^{2})$ for stochastic forcing (A) and a Gaussian distribution $N(1,\sigma_{p}^{2})$ for initial error with a standard deviation of *σ*_*p*_ = 10% (B). Stochastic forcing and initial error lead to similar growth patterns, but initial error exhibits faster growth. The same analysis for PPU is displayed in (C-D). The log-transformed ensemble spread and PPU are averaged over the results from 1,000 synthetic outbreaks. In general, PPU decreases much faster for initial error than stochastic forcing.

We generated 1,000 synthetic outbreaks using Latin hypercube sampling of initial conditions and parameters, with transmission rate forced by daily absolute humidity for New York City, and then imposed perturbations on these trajectories each day from 10 weeks (70 days) prior to the peak until 6 weeks (42 days) after. We measured the log-transformed ensemble spread log(*σ*^2^(*t*,*k*)) averaged over all trajectories for 6 weeks (42 days) following the perturbation. In Fig 1A and 1B, we show the evolution of ensemble spread after perturbations with *σ*_*p*_ = 10% at different times with respect to the outbreak peak.

In general, the growth of uncertainty introduced from stochastic forcing and initial error exhibit qualitatively similar patterns (Fig 1). This finding indicates that the impact of stochastic fluctuation is largely manifested by the nonlinear growth of error it introduces into the model. The stochasticity-induced uncertainty is not static, but will propagate following the nonlinear model dynamics, just as the introduced initial error propagates dynamically. This implies, in generating variability within an ensemble of model trajectories used for influenza forecast, using a stochastic model is equivalent in effect to perturbing initial conditions, but differs in that perturbations from initial conditions (Fig 1B) result in a larger ensemble spread than stochastic fluctuations, which appear to partially damp dynamic error growth (Fig 1A). The impact of these errors depends heavily on both the perturbation time and forecast horizon. Errors introduced before the peak amplify exponentially during the early phase of outbreaks, whereas perturbations after the peak generally remain stable. Other perturbations for *σ*_*p*_ = 5% and 15% were tested (see S1 Text, Figs B-C), but no significant change in the results was observed. Further, we performed the same analysis as in Fig 1 for three other cities with different climate conditions–Denver, Los Angeles and Houston (see S1 Text, Figs D-G). The error growth patterns were robust across these different regions of the US.

Around the peak of an outbreak, a forecast with a large ensemble spread may still have utility because the forecast target also increases. To account for the increased target, we use another measure of predictability, potential prediction utility (PPU) [47,48], to quantify the forecast uncertainty relative to the target. PPU for a prediction made at time *t* with a forecast length *k* is expressed as

$$PPU(t,k)\equiv \frac{1}{1+\sigma (t,k)/\bar{f}(t,k)}.$$ (7)

Recall that *σ*(*t*,*k*) and $\bar{f}(t,k)$ are the ensemble standard deviation and ensemble mean. The term $\sigma (t,k)/\bar{f}(t,k)$ measures the “noise-to-signal” ratio. PPU can vary from one to zero, with a value of one indicating a perfect prediction. In Fig 1C and 1D, the evolution of PPU after perturbation is compared between stochastic fluctuation and initial error. PPU for stochastic forcing remains almost constant at around 0.9, indicating a stable relative uncertainty with respect to the true signal for all perturbations. PPU for initial error, however, has more complex features. Generally, PPU rapidly drops below 0.85 at 7 days after the perturbation, and then continues to decrease at a rate that depends on *t*, the time of perturbation. In Fig 1D, we observe two blue areas with extremely low PPU. The one in the upper-left corner is attributed to the large ensemble spread *σ*(*t*,*k*) produced during the exponential growth of epidemics, while the one in the upper-right corner is due to low signal $\bar{f}(t,k)$ at the end of outbreaks. For days -20 to 0, the large signal, $\bar{f}(t,k)$, near the peak leads to increased PPU values. The same pattern was also observed in experiments for other cities and perturbations with *σ*_*p*_ = 5% and 15%.

From above analyses we conclude that the predictability loss in the SIRS model due to initial error is more pronounced than that from stochastic fluctuation, which is in agreement with findings from climate models [13]. In the next section, we examine the rate and direction of initial error growth.

#### Nonlinear growth of initial error

For this parsimonious 2-dimension ordinary-differential-equation model of influenza transmission, we employ singular vector analysis to estimate the speed and direction of initial error expansion. This method has been applied with great success in numerical weather prediction [49–51].

For the humidity-driven SIRS model (Eqs 1 and 2), we assume that model parameters *R*_0*max*_, *R*_0*min*_, *L* and *D* do not change and define the variable vector ***x*** = (*S*,*I*)^*T*^. We then write Eqs 1 and 2 in the form

$$\frac{dx}{dt}=A(x).$$ (8)

Here ***A***(***x***) is the function describing the nonlinear evolution of the variable vector ***x***. We examine how small perturbations evolve following these nonlinear dynamics. Instantaneous error growth for a small perturbation, *δ****x*** = (*δS*,*δI*)^*T*^, at time *t* is given by the linear system

$$\frac{d\delta x}{dt}=A_{l}\delta x,$$ (9)

where $A_{l}={\frac{dA}{dx}|}_{x(t)}$ is the Jacobian of the system at time *t*:

$$A_{l}=\left(\begin{array}{cc} -\frac{1}{L}-\frac{\beta (t)I}{N} & -\frac{1}{L}-\frac{\beta (t)S}{N}\\ \frac{\beta (t)I}{N} & \frac{\beta (t)S}{N}-\frac{1}{D} \end{array}\right)=\frac{1}{D}\left(\begin{array}{cc} -1/L\prime -I\prime & -1/L\prime -S\prime \\ I\prime & S\prime -1 \end{array}\right).$$ (10)

In the last expression, *S*′ = *R*_0_(*t*)*S*(*t*)/*N*, *I*′ = *R*_0_(*t*)*I*(*t*)/*N*, *L*′ = *L*/*D*. Recall that *R*_0_(*t*) = *β*(*t*)*D* and note that the last matrix in Eq 10 is non-dimensional. Epidemiologically, *S*′ is the rescaled effective reproductive number, i.e., the average number of infections caused by one infection in *D* days in a population with *S*(*t*) susceptible people; *I*′ is the rescaled force of infection, i.e., the hazard (or rate) of a susceptible individual acquiring influenza in *D* days.

In a population of size *N* = 10^5^, the typical error (or uncertainty) in *S* is of order *O*(10^3^), whereas for *I* it is usually of order *O*(10^2^). To give the two errors approximately equal weight we normalize the absolute errors *δS* and *δI* by their typical uncertainties *η*(*S*) and *η*(*I*): $\delta \bar{x}=W\delta x={\left(\delta S/\eta (S),\delta I/\eta (I)\right)}^{T}$ with ***W*** = diag(1/*η*(*S*), 1/*η*(*I*)). For the new variable $\delta \bar{x}$, the error growth equation becomes

$$\frac{d\delta \bar{x}}{dt}=W\frac{d\delta x}{dt}=W\;A_{l}\delta x=W\;A_{l}W^{-1}W\delta x={\bar{A}}_{l}\delta \bar{x},$$ (11)

where, after defining *ν* = *η*(*S*)/*η*(*I*),

$${\bar{A}}_{l}=W\;A_{l}W^{-1}=\frac{1}{D}\left(\begin{array}{cc} -1/L\prime -I\prime & -\nu^{-1}(1/L\prime +S^{\prime })\\ \nu I\prime & S\prime -1 \end{array}\right).$$ (12)

The direction ${\delta \bar{x}(t)=e}_{1}$ that has the fastest instantaneous error growth rate at time *t* is the one that maximizes the quantity

$$\frac{d{\left\|\delta \bar{x}(t)\right\|}^{2}/dt}{{\left\|\delta \bar{x}(t)\right\|}^{2}}.$$ (13)

The norm ‖***x***‖^2^ is defined as ‖***x***‖^2^ = ***x***^*T*^***x***. In Eq 13, the numerator $d{\left\|\delta \bar{x}(t)\right\|}^{2}/dt$ quantifies the instantaneous growth rate of ${\left\|\delta \bar{x}(t)\right\|}^{2}$ (square of the Euclid length of $\delta \bar{x}(t)$). The denominator normalizes this growth rate by ${\left\|\delta \bar{x}(t)\right\|}^{2}$. Therefore, Eq 13 represents the relative instantaneous growth rate of a perturbation $\delta \bar{x}(t)$. If we consider unit perturbations with ${\left\|\delta \bar{x}(t)\right\|}^{2}=1$, the growth rate is solely determined by $d{\left\|\delta \bar{x}(t)\right\|}^{2}/dt$.

Because, by Eq 11

$$\frac{d({\delta \bar{x}}^{T}\delta \bar{x})}{dt}={\delta \bar{x}}^{T}\frac{d\delta \bar{x}}{dt}+\frac{d{\delta \bar{x}}^{T}}{dt}\delta \bar{x}={\delta \bar{x}}^{T}({\bar{A}}_{l}+{{\bar{A}}_{l}}^{T})\delta \bar{x},$$ (14)

the direction ***e***_1_ that grows the fastest is the solution of the eigenvalue problem

$$({\bar{A}}_{l}+{{\bar{A}}_{l}}^{T})e_{1}=\lambda_{1}e_{1}.$$ (15)

The largest eigenvalue (the fastest growth rate) *λ*_1_ may be found analytically:

$$D\lambda_{1}=S^{\prime }-I^{\prime }-1-1/L\prime +\sqrt{{\left(I^{\prime }+S\prime -1+1/L\prime \right)}^{2}+{\left(\nu^{-1}/L\prime +\nu^{-1}S^{\prime }-\nu \;I\prime \right)}^{2}}.$$ (16)

The principal eigenvector ***e***_1_ is called the singular vector of the system [48]. It is an approximation of the local Lyapunov vector [52–54]. Note that the singular vector is different from the principal eigenvector of the Jacobian ${\bar{A}}_{l}$. The impact of each variable or parameter on the (non-dimensional) error growth rate *Dλ*_1_ can be calculated from Eq 16. Since *L* ∈ [1,10] years ≫ *D* ∈ [2,7] days, we will omit the term 1/*L*′ = *D*/*L* hereafter.

**Fig 2:**
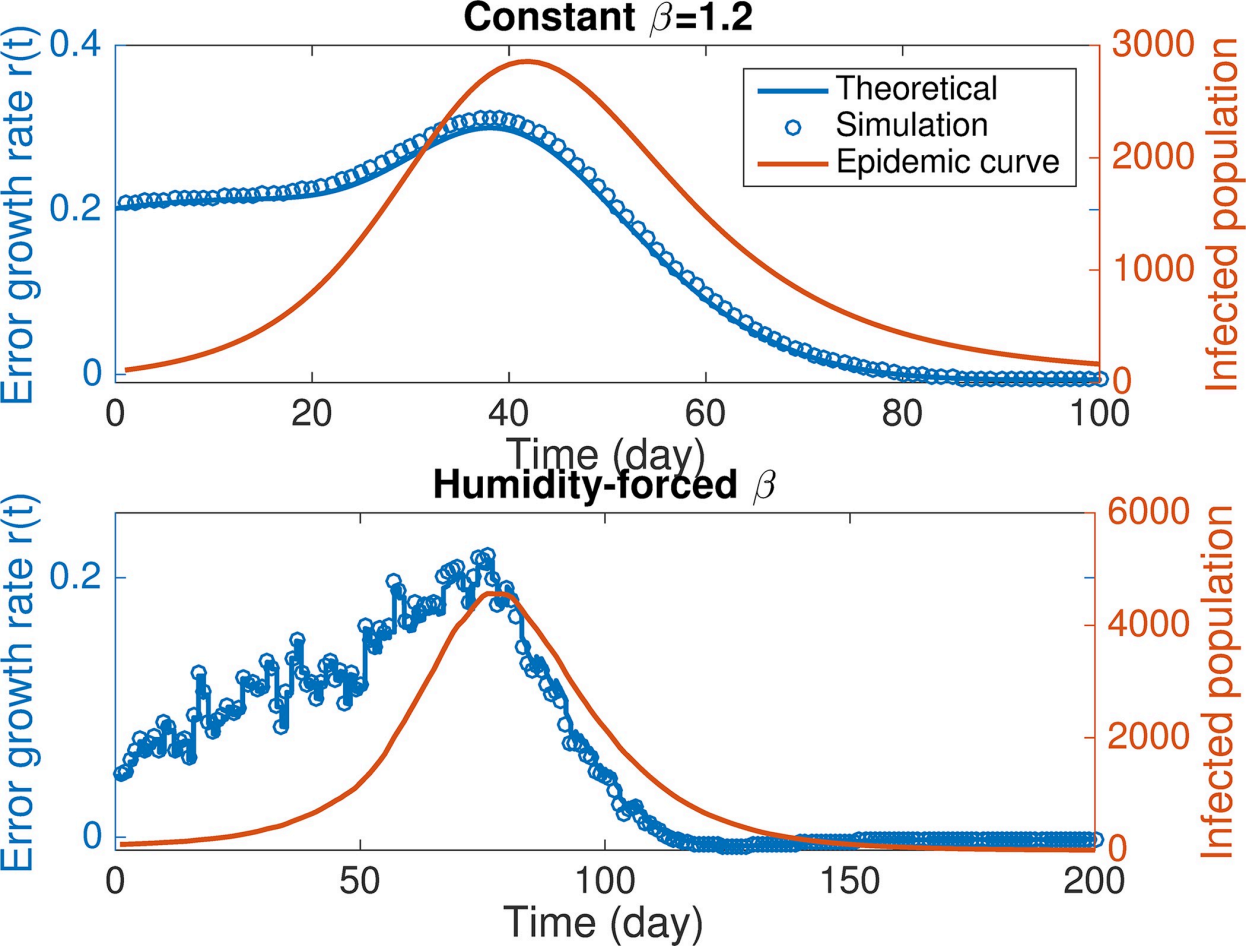
Comparison of the simulated and theoretical values of the maximal error growth rate. For SIRS models with constant *β* = 1.2 (A) and humidity forced *β* (B), we compare the maximal error growth rate at different phases in an outbreak as predicted by Eq 16 and calculated from simulations. The initial condition and parameters in A are set as *N* = 10^5^, *S* = 0.5 × 10^5^, *I* = 100, *β* = 1.2, *L* = 730 days and *D* = 5 days. B uses the setting *N* = 10^5^, *S* = 0.5 × 10^5^, *I* = 100, *R*_0*max*_ = 3.5, *R*_0*min*_ = 1.2, *L* = 730 days and *D* = 5 days, where *β* is forced by daily absolute humidity for New York City starting from October 1^st^. The x-axis shows the time (day) after the beginning of model integration. Errors in *S* and *I* are normalized by *η*(*S*) = 10^3^ and *η*(*I*) = 10^2^. The red line shows the simulated outbreak as reference. In both cases, the simulated error growth rates are well predicted by their theoretical values.

To validate Eq 16, we calculated the maximal error growth rate numerically and then compared it with the theoretical value. At each day *t* after the beginning of an outbreak, we imposed an ensemble of perturbations on ***x*** along different directions in the (*S*,*I*) plane: *δ****x*** = (cos(2*kπ*)*η*(*S*), sin(2*kπ*)*η*(*I*))^*T*^ (*k* = 1/360,⋯, 1, *η*(*S*) = 10^3^, *η*(*I*) = 10^2^) $(\delta \bar{x}={\left(\cos\left(2k\pi \right),\sin\left(2k\pi \right)\right)}^{T}$ in the normalized space). Both the unperturbed and perturbed trajectories were evolved forward for *δt* = 0.1. We then calculated the error at *t* + *δt* and the maximal error growth rate among all perturbations according to Eq 13. In Fig 2, we compare the numerically calculated maximal error growth rate *r*(*t*) with that predicted by Eq 16 for the SIRS models with or without humidity forcing. In both cases, the maximal error growth rate is well predicted by Eq 16. Further, according to the overlaid epidemic curves, error growth is most pronounced at the early stage of outbreaks, indicating that model dynamics are more sensitive to the errors introduced early in the season. We repeated this analysis for 1,000 synthetic outbreaks, and display the distribution of discrepancy between theoretical and simulated error growth rate in S1 Text and Fig H. Results indicate a satisfactory performance from the theoretical prediction of Eq 16.

**Fig 3:**
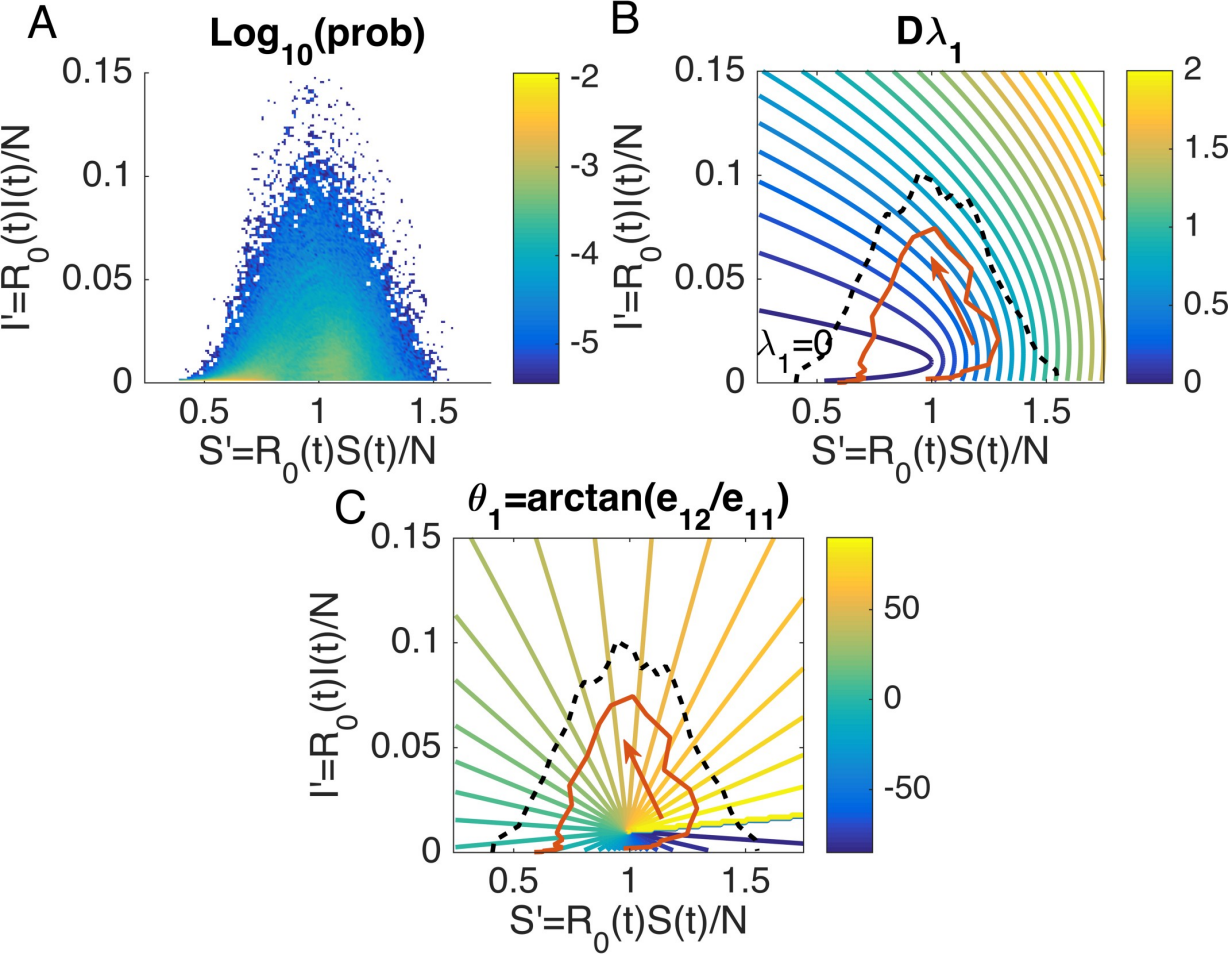
Impact of model parameters on the maximal error growth rate. (A) Distribution of *S*′ = *R*_0_(*t*)*S*(*t*)/*N* and *I*′ = *R*_0_(*t*)*I*(*t*)/*N* for 1,000 synthetic outbreaks. The color shows the logarithmic probability (base 10) derived from synthetic outbreaks, forced by humidity conditions for New York City, for 280 days (40 weeks) after October 1^st^. (B) The contour of *Dλ*_1_ as a function of *S*′ and *I*′ is generated from Eq 16, in which the parameter *L*′ is omitted due to its nominal effect. Errors are normalized by *η*(*S*) = 10^3^ and *η*(*I*) = 10^2^. *Dλ*_1_ quantifies the error growth rate given a certain infectious period *D*. Contour lines correspond to *Dλ*_1_ values ranging from 0 (blue) to 2 (yellow) with an interval of 0.1. The contour line corresponding to *λ*_1_ = 0 is highlighted. The black dashed line marks the feasible region of (*S*′,*I*′) for synthetic outbreaks with probability higher than 10^−5^ in (A). The red curve shows the trajectory of one particular outbreak in the *S*′ − *I*′ plane. As the outbreak unfolds, the error growth rate first increases and then gradually decreases, implying an increased ensemble spread in the forecast system attributable to the model dynamics near the outbreak peak. (C) The contour of *θ*_1_ = *arctan*(*e*_12_/*e*_11_) (in degree from −90° to 90°) that represents the direction of the eigenvector ***e***_1_ = (*e*_11_,*e*_12_)^*T*^ corresponding to *λ*_1_. The x-coordinate *e*_11_ and y-coordinate *e*_12_ represent the projections of ***e***_1_ on *S*′ and *I*′, respectively. Contour lines indicate values from −90° (blue) to 90° (yellow) with an interval of 5°. During the epidemic process marked by the red curve, θ_1_ first changes from around −40° to −90°, and then from 90° to 0°. This suggests that the fastest error growth direction moves to align with *I*′ (*e*_12_ > *e*_11_) during the first stage of the outbreak and then gradually turns to *S*′ (*e*_11_ > *e*_12_).

To identify realistic combinations of (*S*′,*I*′), we generated 1,000 synthetic outbreaks using the SIRS model forced by humidity conditions for New York City starting from October 1^st^. The distribution of *S*′ and *I*′ in the (*S*′,*I*′) plane, calculated from these synthetic outbreaks over 280 days (40 weeks), is shown in Fig 3A. We display the contour of *Dλ*_1_ as a function of *S*′ and *I*′ in Fig 3B (*η*(*S*) = 10^3^, *η*(*I*) = 10^2^). The area contained by the black dashed line marks the region of (*S*′,*I*′) in Fig 3A with probability of occurrence higher than 10^−5^. In this feasible region, *Dλ*_1_ is quite sensitive to *S*′ but less sensitive to *I*′ such that the error growth rate depends primarily on the size of the susceptible population. Epidemiologically, this indicates that the uncertainty of future, predicted incidence is more strongly linked to the proportion of susceptible people in the population than to the proportion of infected individuals. For each particular outbreak, we can draw its trajectory in the *S*′ − *I*′ plane and observe how the growth rate changes over time (see the red trajectory in Fig 3B for an example).

The fastest error growth direction can be estimated by the eigenvector ***e***_1_ = (*e*_11_,*e*_12_)^*T*^ corresponding to *λ*_1_. We quantify the direction of ***e***_1_ by *θ*_1_ = *arctan* (*e*_12_/*e*_11_) (in degrees from −90° to 90°), and show its contour in Fig 3C. In the middle of the feasible region is a singular point where ${\bar{A}}_{l}+{{\bar{A}}_{l}}^{T}$ degenerates to *diag*(0,−0.02). In fact, the singular point is the vertex of the parabola of *Dλ*_1_ = 0 defined by Eq 16 (Fig 3B). At this point, we have ***e***_1_ = (0,1)^*T*^ where *e*_12_/*e*_11_ diverges. An epidemic could reach this singular point. This would lead to the divergence of *θ*_1_ around this point but would not affect the epidemic process described by Eqs 1 and 2.

During the epidemic process marked by the red curve in Fig 3C, *θ*_1_ first changes from approximately −40° to −90°, and then from 90° to 0°. Note that ***e***_1_ and −***e***_1_ (the opposite of ***e***_1_) are both eigenvectors. Thus, the directions between −90° and 0° are equivalent to their opposite directions between 90° and 180°. In this sense, the fastest error growth direction evolves continuously from 140° to 0°. Recall that *e*_11_ and *e*_12_ represent the projections of ***e***_1_ on *S*′ and *I*′, respectively. This implies, in the normalized space, the error growth direction gradually moves to align with *I*′ (*e*_12_ > *e*_11_) at the early phase and then turns to *S*′ (*e*_11_ > *e*_12_) in the end.

Fig 3 provides a simplified picture to interpret the impact of parameters on error growth. According to Eq 16, the second eigenvalue *λ*_2_ is always negative. Therefore, errors along the direction of the eigenvector corresponding to *λ*_2_ will always contract, and the only concern is for error growth along ***e***_1_. The growth rate and direction of these errors are described in Fig 3B and 3C. Varying *D* changes the time scale of error growth; changing *R*_0_ modifies the position of (*S*′,*I*′) in the (*S*′,*I*′) plane by a given scaling parameter. The evolution of error growth for an outbreak can be tracked in a trajectory in the (*S*′,*I*′) plane, as plotted in Fig 3B and 3C.

**Fig 4:**
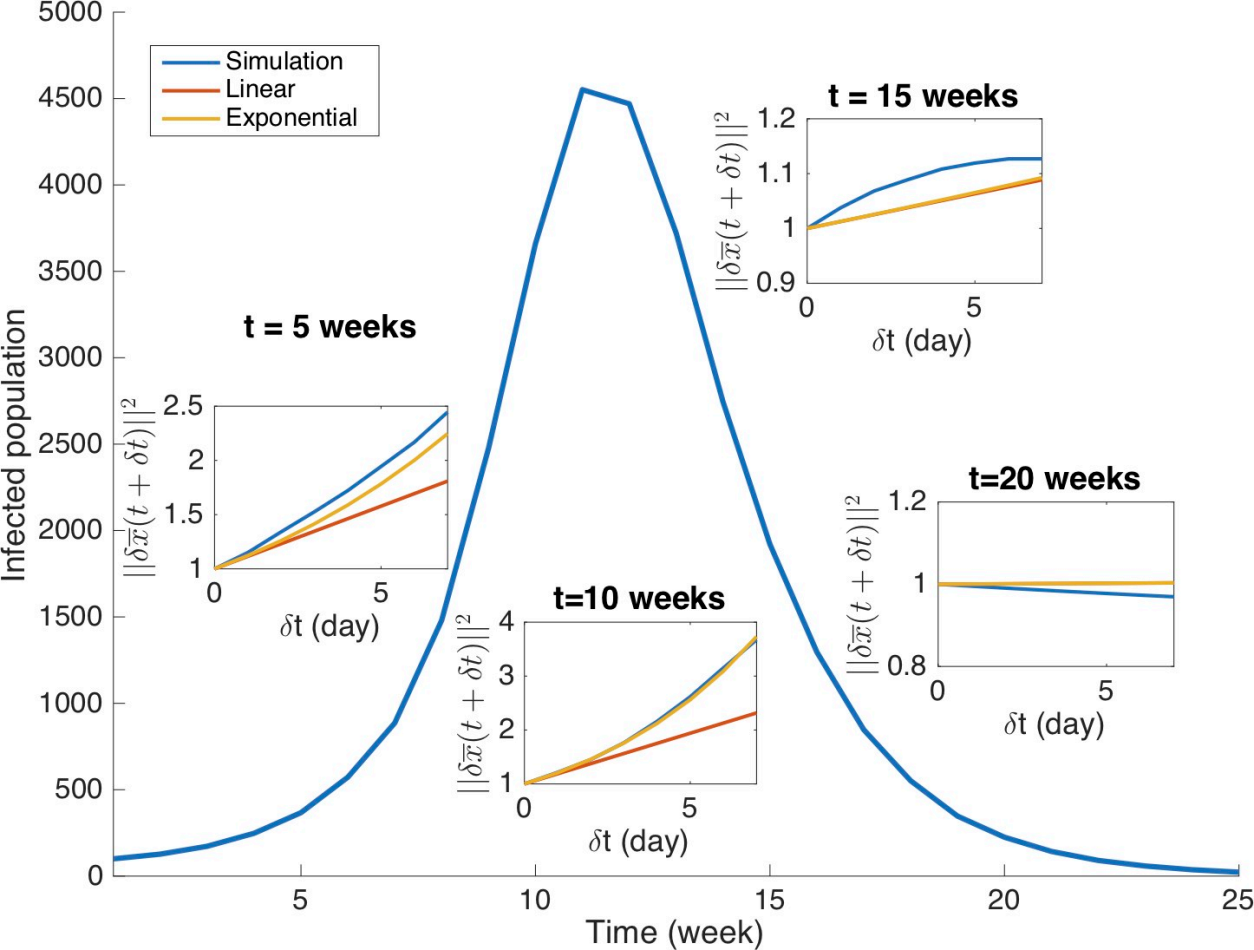
Approximation of the nonlinear error growth at different phases. We inspect whether nonlinear error growth can be approximated by the linearized system at different stages of the outbreak. Starting from *t* = 5 weeks, we display the error ${\left\|\delta \bar{x}(t+\delta t)\right\|}^{2}$ in the following week obtained from simulation with full nonlinearity (blue lines), and compare it with approximations using a linear extrapolation $(1+\lambda_{1}\delta t){\left\|\delta \bar{x}(t)\right\|}^{2}$ (red lines) and an exponential growth $\exp(\lambda_{1}\delta t){\left\|\delta \bar{x}(t)\right\|}^{2}$ (yellow lines). The initial error is set as ${\left\|\delta \bar{x}(t)\right\|}^{2}=1$. The growth rate *λ* is calculated from Eq 20, where *η*(*S*) = 10^3^ and *η*(*I*) = 10^2^. The same analyses at *t* = 10, 15 and 20 weeks are shown in other insets. The epidemic curve is generated from the SIRS model forced by the humidity condition for New York City starting from October 1^st^. The exponential approximation agrees well with the simulated error at 5 and 10 weeks, whereas the linear approximation is only valid within a short time period. At weeks 15 and 20 (after peak), both the exponential and linear approximations give satisfactory estimates of the nonlinear simulation.

As the error growth in the dynamical model is intrinsically nonlinear, it may deviate from the linear approximation characterized by the matrix ${\bar{A}}_{l}+{{\bar{A}}_{l}}^{T}$. By using a linearized system to study error growth, we assume that the linear approximation generally captures the behavior of the full nonlinear system within a certain time interval. To verify this assumption, it is important to quantify the deviation of the linear approximation from the full nonlinear system. In Fig 4, we compare the error growth in the nonlinear system with approximations at four different phases of an outbreak (*t* = 5, 10, 15, and 20 weeks). At each time point *t*, we added an ensemble of errors *δ****x*** = (cos(2*kπ*)*η*(*S*), sin(2*kπ*)*η*(*I*))^*T*^ (*k* = 1/360,⋯, 1, *η*(*S*) = 10^3^, *η*(*I*) = 10^2^) (equivalently, $\delta \bar{x}={\left(\cos\left(2k\pi \right),\sin\left(2k\pi \right)\right)}^{T}$ in the normalized space) to the variables and bred the errors for 7 days. We display the largest error ${\left\|\delta \bar{x}(t+\delta t)\right\|}^{2}$ after *δt*, and compare it with two approximations: 1) a linear extrapolation $(1+\lambda_{1}\delta t){\left\|\delta \bar{x}(t)\right\|}^{2}$, and 2) an exponential growth $\exp\left(\lambda_{1}\delta t\right)\;{\left\|\delta \bar{x}(t)\right\|}^{2}$ for ${\left\|\delta \bar{x}(t)\right\|}^{2}=1$. Here *λ*_1_ is the largest eigenvalue of the linear propagator ${\bar{A}}_{l}+{{\bar{A}}_{l}}^{T}$. As shown in Fig 4, the exponential approximation provides a good agreement with the full nonlinear growth at the early stage, indicating that the error will grow exponentially with a rate *λ*_1_. The linear approximation, however, is only valid for small *δt* and tends to underestimate the error growth after 2 days, especially before the outbreak peak. The largest eigenvalue *λ*_1_, although obtained from a linearized system, can reliably quantify the speed of nonlinear error growth between two successive observations.

#### Applications in conjunction with the EAKF

**Fig 5:**
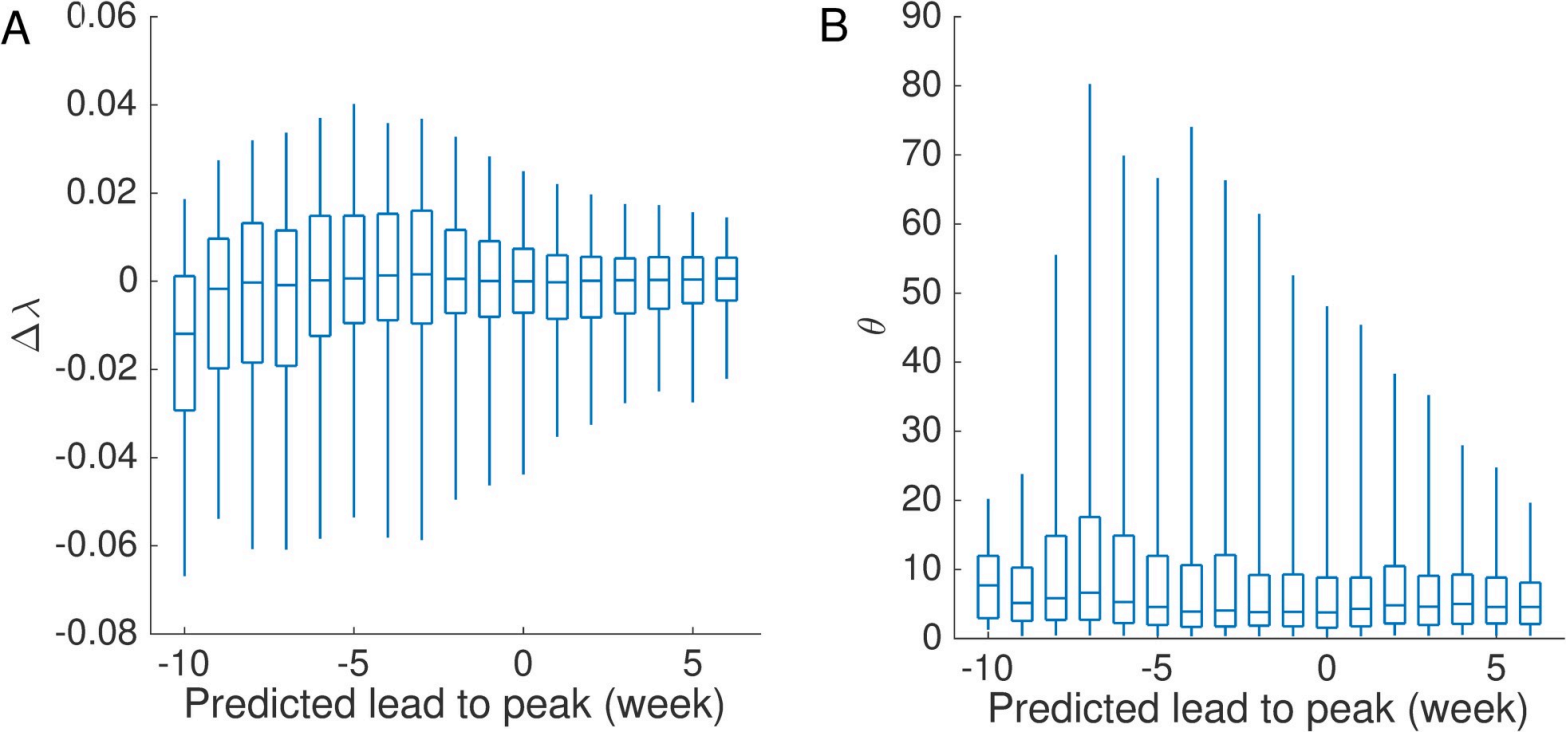
Estimation bias in error growth rate and direction using the EAKF for synthetic outbreaks. We use the EAKF to infer model parameters and variables for 1,000 different synthetic outbreaks generated using the humidity-driven SIRS model, and calculate the error growth rate ${\hat{\lambda }}_{1}$ and direction ${\hat{e}}_{1}$. Normalization denominators *η*(*S*) and *η*(*I*) are set as the 95 percentile of ensemble members’ deviations from ensemble mean. Distributions of the estimation bias in error growth rate $\Delta \lambda ={\hat{\lambda }}_{1}-\lambda_{1}$ and the angular deviation from ${\hat{e}}_{1}$ to ***e***_1_ (in degree from 0° to 90°) $\theta =\arccos\left({\hat{e}}_{1}^{T}e_{1}\right)$ are reported in (A-B). The boxes and whiskers indicate the interquartile and the minimal and maximal values. The x-axis indicates the relative forecast time with respect to the predicted peak, i.e., forecast week minus predicted peak week. A negative predicted lead indicates the peak is predicted to occur in the future, whereas a positive lead indicates the peak is predicted to have already passed. For all predicted leads to peak, the deviation of the error growth rate, *λ*_1_, is distributed around 0, and the angular deviation of ***e***_1_ is mostly below 10°. As a result, the error growth rate and direction estimated using the EAKF can be used to generate perturbations of the forecast system.

The above analyses are performed on the assumption that model parameters and variables are known. In an operational forecast, unobserved parameters and variables can be estimated using data assimilation techniques. In this work, we use the ensemble mean of parameters and variables obtained using the EAKF to calculate the matrix ${\bar{A}}_{l}+{{\bar{A}}_{l}}^{T}$. Error normalization denominators *η*(*S*) and *η*(*I*) are set as the 95 percentile of ensemble member distance to the ensemble mean so that most errors fall within the unit circle. Outliers are not considered due to their large variation. In order to inspect the estimation bias in error growth rate *λ*_1_ and direction ***e***_1_, we ran the SIRS-EAKF system with *n* = 300 ensemble members for 1,000 synthetic outbreaks for which the actual *λ*_1_ and ***e***_1_ can be calculated, and computed the estimated ${\hat{\lambda }}_{1}$ and ${\hat{e}}_{1}$ in 40 consecutive weeks. In Fig 5A, we display the distribution of estimation bias in error growth rate $\Delta \lambda ={\hat{\lambda }}_{1}-\lambda_{1}$, grouped by the predicted lead to peak ranging from -10 weeks to 6 weeks (a negative predicted lead indicates the peak is predicted to occur in the future; a positive lead indicates the peak is predicted to have already passed). The boxes and whiskers indicate the interquartile and the minimal and maximal values. In general, *Δλ* is distributed around 0 within a small range, suggesting that the error growth rate *λ*_1_ can be well estimated. The bias in ***e***_1_ is quantified by the angular deviation from ${\hat{e}}_{1}$ to ***e***_1_ (in degree from 0° to 90°) $\theta =\arccos\left({\hat{e}}_{1}^{T}e_{1}\right)$. The distributions of *θ* are shown in Fig 5B. The estimation bias *θ* is low for the majority of cases. As a result, the estimated ${\hat{e}}_{1}$ generally has a large projection on the actual ***e***_1_.

### Retrospective forecast of historical influenza outbreaks

#### Optimal perturbation for ensemble forecasts

As in numerical weather and climate prediction, information on error growth can be harnessed to improve the forecast quality of the model-data assimilation system. In principle, perturbations along the fastest error growth direction, termed optimal perturbations [4], are imposed when the ensemble spread needs to be enlarged to account for uncertainty in targets. Specifically, for each ensemble member, we adjust the component of $\delta \bar{x}={\left(\delta S/\eta (S),\delta I/\eta (I)\right)}^{T}$ along the estimated ${\hat{e}}_{1}$ by a factor *k*: $(\delta {\bar{x}}^{T}{\hat{e}}_{1}){\hat{e}}_{1}\to k(\delta {\bar{x}}^{T}{\hat{e}}_{1}){\hat{e}}_{1}$, and use the adjusted variables to project the model ensemble into the future to make forecast. Model parameters are not adjusted. The deviations *δS* and *δI* are obtained from the difference between the ensemble member and ensemble mean. If *k* > 1, the perturbation expands the distribution of variables along ${\hat{e}}_{1}$ in the normalized space. Since the variability of incidence and dynamical error growth rate changes over time, we assign different perturbation intensities at different predicted lead to peak.

To determine the perturbation intensity *k* needed for each predicted lead, we optimized *k* to improve the forecast quality of near-term predictions, here meaning the forecast of incidence in the next one to four weeks ahead. The quality of probabilistic forecasts can be measured using a reliability plot [55]. We divide the forecast range into 14 categories: [0,1 × 10^3^),⋯,[1.2 × 10^4^, 1.3 ×10^4^), [1.3 × 10^4^, ∞) (infections per 10^5^ people). For a large number of forecasts, we can calculate the probability of falling into each category *P*_*pred*_(*i*), averaged over the full ensemble distribution of multiple forecasts, as well as the actual observed frequency of occurrence in each category *P*_*occur*_(*i*). The 14 points (*P*_*pred*_(*i*),*P*_*occur*_(*i*)) form the reliability plot. A perfect probabilistic forecast satisfies *P*_*pred*_(*i*) = *P*_*occur*_(*i*) for 1 ≤ *i* ≤ 14. In the reliability plot, this means all 14 points fall on the diagonal line *y* = *x*. Here, we use the deviation of the points from the diagonal line ∑_*i*_|*P*_*pred*_(*i*) − *P*_*occur*_(*i*)| to quantify the forecast quality. Our objective is to minimize the average deviation for lead times of one to four weeks over predictions from -8 to 6 weeks relative to the predicted peak.

We optimized the perturbation intensity using simulated annealing [56] (see details in S1 Text, Fig I). To give a fair evaluation of the perturbation procedure, half of historical outbreaks in 95 US cities during the 2003–2004 through 2013–2014 seasons (excluding the 2008–2009 and 2009–2010 pandemic seasons) were used in the optimization, and the other half were used in out-of-sample validation. The historical outbreaks selected for training and validation are reported in S1 Text (Table A). (The Matlab code and data for retrospective forecast are provided in S1 Code). To understand the baseline behavior of the SIRS-EAKF system, we display the reliability plots for 1- to 4-week prediction in S1 Text, grouped by the predicted lead to peak. In general, reliability plots have a greater deviation from the diagonal line at predicted lead between 0 to 6 weeks (Figs J-M).

**Fig 6:**
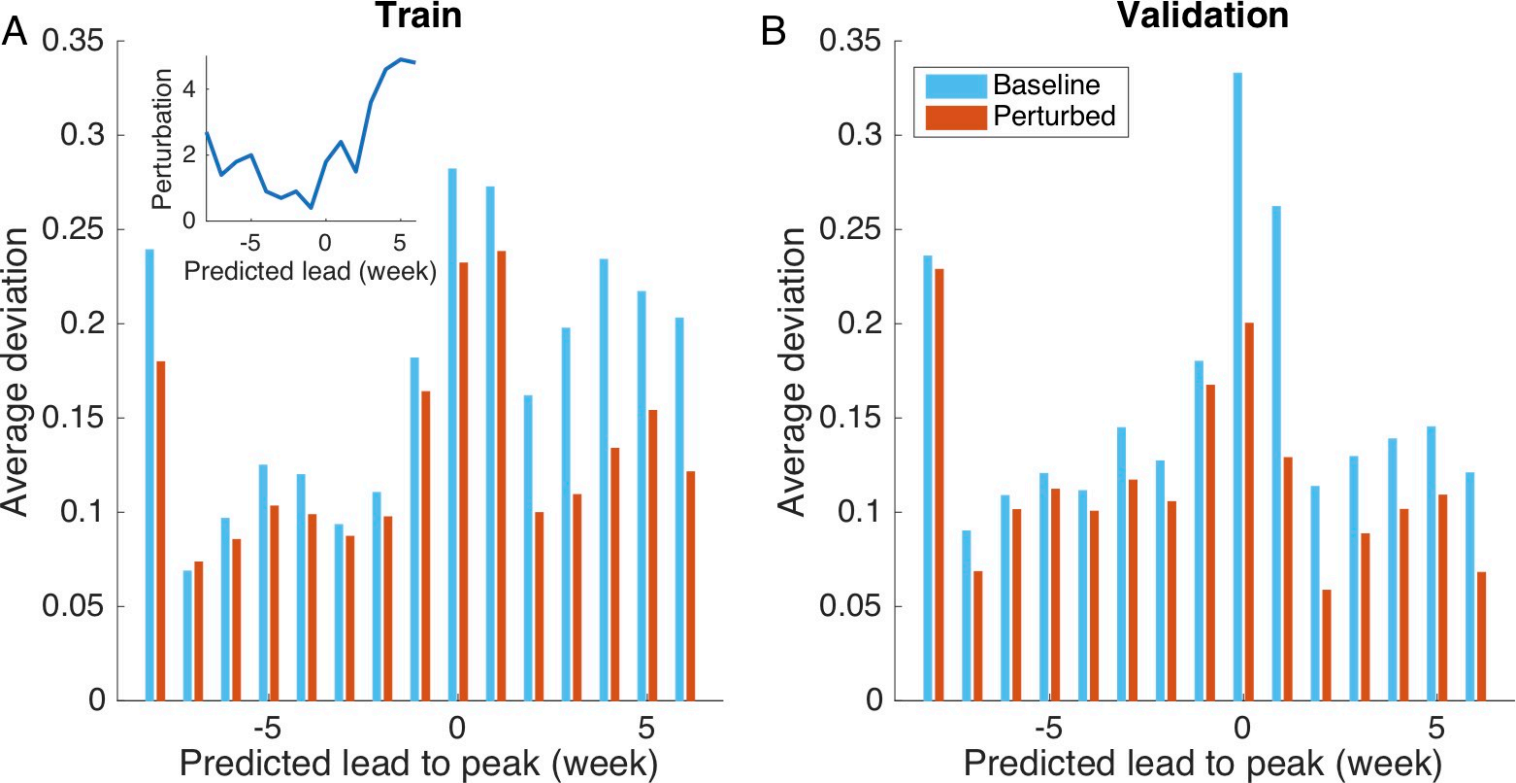
Reduction of deviation in reliability plot achieved by perturbation in retrospective forecast. We use half of historical outbreaks in 95 US cities during the 2003–2004 through 2013–2014 seasons (excluding the 2008–2009 and 2009–2010 pandemic seasons) to optimize the perturbation intensity in which the deviation of the reliability plot is minimized. Inset shows the optimized perturbation strategy. The comparison of average deviation for baseline and perturbed SIRS-EAKF systems is presented in (A). We validate the perturbation procedure using the other half of historical outbreaks, and report the comparison of average deviation in (B). For both training and validation data, the perturbation procedure (red bars) reduces reliability plot deviation, particularly for predicted leads between 0 and 6 weeks.

In Fig 6A, the reduction of deviation in the reliability plot is shown for different predicted leads. The deviation in the reliability plot (y-axis) is averaged over 4 targets, i.e., 1- to 4-week predictions. Figures breakdown for each target are shown in S1 Text (Figs N-O). Improvement is most pronounced around and after the peak. The inset shows the optimized perturbation intensity *k*. According to the optimization, perturbations have roughly three phases: 1) -8 to -5 weeks. Errors have a slow growth at the early stage of an outbreak. Therefore, the ensemble spread needs to be expanded (*k* > 1). However, since the targets remain low without too much variation, this expansion should not be too large. 2) -4 to -1 week. Errors can expand exponentially during the rapid growth of an outbreak. The dynamical expansion alone is enough to generate ensemble spread. No additional expansion is needed (*k* ≈ 1). 3) After 0 week. The error growth rate becomes lower after the peak where targets drop fast from high to low values. A strong expansion is needed to supplement the ensemble spread and capture the large variation in targets.

To validate the perturbation procedure, we ran retrospective forecasts for the rest of the historical outbreaks using the optimized perturbation intensity. Weekly forecasts of incidence during the next one to four weeks were generated. In Fig 6B, we compare the average deviation in the reliability plot for these 4 targets between the baseline (without perturbation) and the perturbed system. Forecasts are improved as in the training data set (Fig 6A), indicating there is no over-fitting issue.

**Fig 7:**
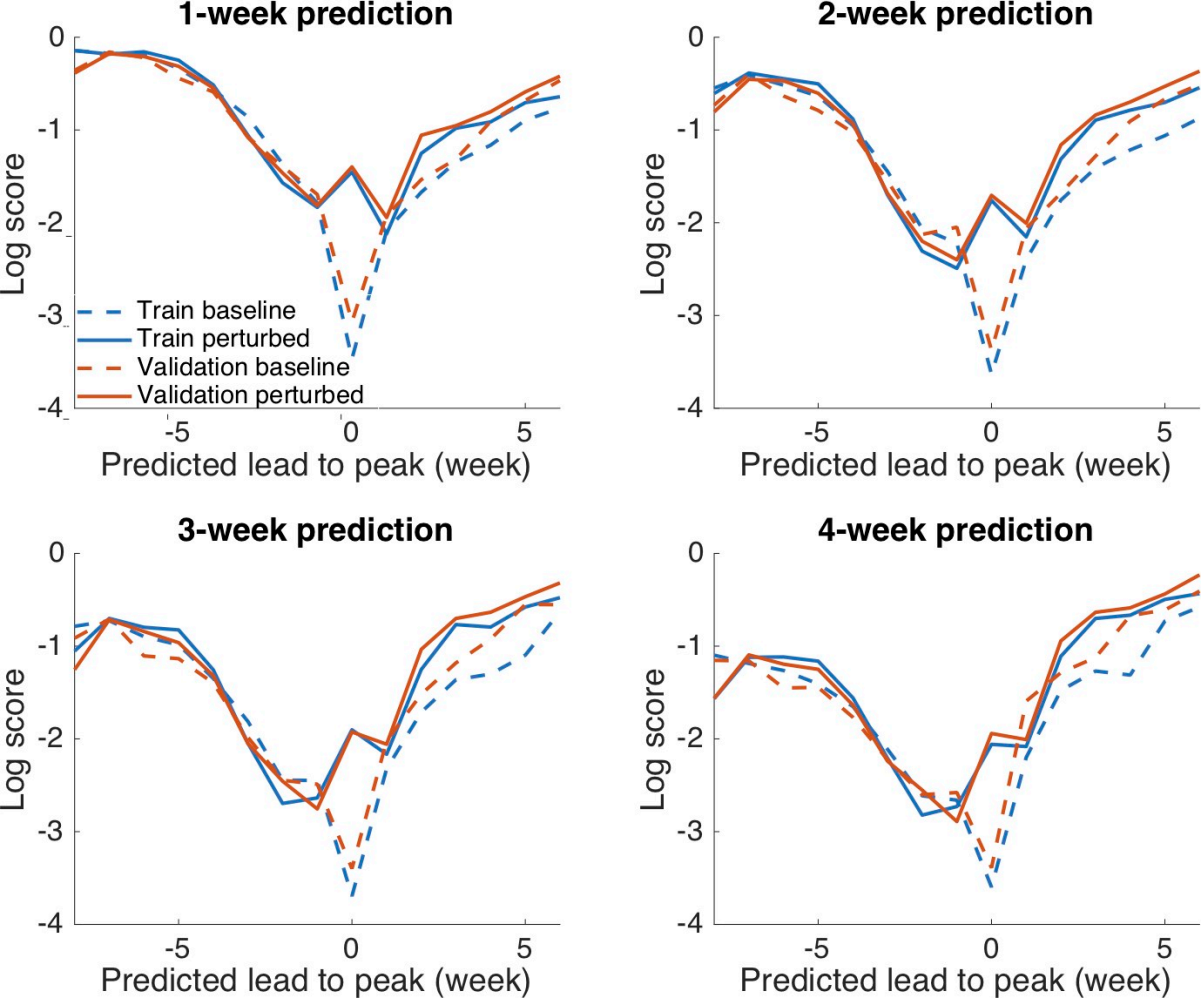
Log scores from training and out-of-sample retrospective forecast using the baseline and perturbed SIRS-EAKF systems. Results are averaged for weekly forecasts for a randomly chosen half (training) and the rest (validation) of historical outbreaks in 95 US cities during the 2003–2004 through 2013–2014 seasons, excluding the 2008–2009 and 2009–2010 pandemic seasons. The perturbation procedure improves log scores for all four targets, predominantly at the predicted leads between 0 to 6 weeks.

We also used the “log score” to assess the forecast accuracy. For each forecast target, the *n* = 300 ensemble trajectories are grouped into 14 bins as defined before. The fraction of trajectories falling in each bin *i* is the corresponding predicted weight *w*_*i*_. If the observed target falls in bin *h*, the log score L for a given forecast is defined as the logarithmic value (base *e*) of the weight in bin *h*: $L=\log\left(w_{h}\right)$. If the log score is below -10, we use the floor value of -10. Similar score measures have been used in the US Centers for Disease Control and Prevention's real-time influenza forecast challenge [2,3]. (In S1 Data, we provide the forecast results for the baseline and perturbed EAKF in the format of the influenza forecast challenge.) In Fig 7, we compare the log scores of 1- to 4-week forecasts grouped by predicted lead. Comparison of the log scores obtained from the baseline and perturbed SIRS-EAKF forecasts indicates that the perturbation procedure improves short-term forecast accuracy for historical outbreaks, particularly for forecasts generated near and after peak, i.e., after -1 week. This improvement, observed for both training and out-of-sample seasons, substantially enhances the forecast quality near the peak, where the prediction task is the most challenging. In S1 Text, we report the 5%, 25%, 50%, 75% and 95% percentiles of log scores at each predicted lead to peak for 1- to 4-week prediction (Table B). In general, the perturbation procedure dramatically improves the 5% percentile scores (i.e., bad predictions) at predicted leads between 0 and 6 weeks.

## Discussion

In this work, we show that within a humidity-driven compartmental model used for influenza forecast, the error introduced from initial conditions grows faster than error derived from stochastic fluctuations when these errors are of roughly the same magnitude. For other infectious diseases with lower incidence, however, stochastic effects may play a more crucial role determining the predictability of model dynamics [29–35,57].

In the application of optimal perturbations presented here, we make use of the nonlinear growth of initial error to expand the ensemble spread. This procedure is demonstrated to be effective in enhancing short-term forecast quality by inflating the distribution of ensemble members along the fastest error growth direction. As a consequence, the efficiency of each ensemble member is improved because the perturbed ensemble can explore a larger region of state-space. This implies, for a certain level of forecast accuracy, forecast systems with perturbations would require a smaller number of ensembles. For high-dimensional forecast systems that involve large numbers of localities, such as the system developed in Ref. [6], it should be possible to generate a similar perturbation procedure that reduces ensemble size and thus computational burden.

The mechanistic epidemic model employed here is mis-specified–i.e. it does not represent the full complexity of influenza transmission as it occurs in the real world. For a mis-specified model, initial conditions must be well constrained or error growth will likely deteriorate long-term predictions. If too large, such initial condition error in a mis-specified model will produce unrealistic trajectories that are outside the scope of the real world. (Forecasts generated using a better-specified model also require well-constrained initial conditions; however, the issue of improper initial conditions is more problematic for more grossly mis-specified models, as data assimilation becomes less effective due to the increasing model flaws.) Data assimilation, such as with the EAKF, is a means of partially handling the effects of both model mis-specification and state space error; however, data assimilation methods do not address dynamical error growth. In a recent related work [58], we explored initial condition error growth using a numerical technique–the breeding method–and proposed a method to counteract unrealistic errors growth in the SIRS model. We diagnosed the error structure between unobserved variables and observations using the breeding method, and then examined the deviation of the prediction from observations to further constrain the system using that error growth structure [58]. This error correction procedure does not necessarily reduce the spread of ensemble trajectories or variable/parameter distributions, but does in effect calibrate unrealistic trajectories toward realistic regions in the state space under the assumption that the SIRS model can reasonably well describe the transmission dynamics.

Both optimal perturbation and error correction make use of error growth in the dynamical model; however, the two approaches employ different techniques and perceive the role of error growth from opposite perspectives. First, optimal perturbation examines the *linearized* system in a short time period and uses an *analytical* singular vector analysis to find the fastest error growth direction; whereas in error correction, the error structure is diagnosed using a *numerical* method–the breeding method–which fully preserves the *nonlinear* dynamics. Second, in optimal perturbation, the error growth is *beneficial* to short-term forecast because it increases the spread of prediction; however, in error correction, the error growth is *detrimental* for unrealistic trajectories so that it should be counteracted to calibrate those trajectories toward reasonable regions in the state space. The latter error correction improves the forecast of seasonal targets, e.g., peak week, peak intensity and attack rate. A systematic comparison between optimal perturbation and error correction is needed; however, this task is nontrivial and goes beyond the scope of this study.

The approach presented here does not address model mis-specification but instead uses singular vector analysis to develop optimal perturbations of the ensemble that improve forecast accuracy. The findings indicate that, even for prediction using a simple SIRS model, forecast accuracy can be heavily impacted by factors such as system initialization, ensemble spread, model nonlinearity and error structure. Our challenge going forward is to design operational forecasting systems that optimize and balance all these factors.

## Supporting information

S1 Text Supplementary materials. (DOCX)

S1 Code The Matlab code for the perturbed EAKF and ILI+ data for 95 cities in seasons from 2003 to 2013. (ZIP)

S1 Data Forecast results from the baseline and perturbed EAKF for 1- to 4-week predictions, in the format of the CDC influenza forecast challenge. (ZIP)
