# Supplementary material for: Predictability in process-based ensemble forecast of influenza
Source: PLoS Comput Biol. 2019 Feb 28;15(2):e1006783. doi: 10.1371/journal.pcbi.1006783 (PMC6394909; doi:10.1371/journal.pcbi.1006783)
Supplement: S1 Text — (DOCX) [file pcbi.1006783.s001.docx]

Historical outbreak fitting using the SIRS-EAKF system

The SIRS-EAKF system can flexibly simulate trajectories of historical outbreaks. To demonstrate this, in Figure A, we fitted ILI+ curves from New York, Denver, Los Angeles and Houston for 2010 to 2013. Despite use of a parsimonious SIRS model, the posterior tracks the behavior of historical epidemic curves due to successive adjustment of the state space and parameters during data assimilation.

Perturbation sensitivity tests

In addition to Figure 1 in the main text, we performed tests with different magnitudes of perturbation. In Figures B-C, we repeat the same analysis as in Figure 1 but use alternate standard deviations for the perturbation: 5% and 15%. The resulting error growth patterns are qualitatively similar to that shown in the main text.

We further examined error growth in other three cities with different humidity conditions – Denver, Los Angeles and Houston (see their daily absolute humidity in Figure D). Results in Figures E-G remain similar to those in Figure 1, indicating that error growth patterns are robust across different climate conditions in the US.

Discrepancy between theoretical and simulated error growth rates

To further validate the theoretical prediction of error growth rate by Eq. 16, we generated 1,000 synthetic outbreaks forced by the daily absolute humidity for New York City, and calculated both theoretical and simulated error growth rates for 200 consecutive days. The distributions of discrepancy between theoretical and simulated values are shown in the upper panel of Figure H. Compared with the simulated values displayed in the lower panel of Figure H, the discrepancy in upper panel is small, indicating a satisfactory performance of the theoretical prediction (Eq. 16).

Optimization of perturbation intensity using simulated annealing

For each predicted lead to peak ranging from -8 weeks to 6 weeks, we assume the perturbation intensity $k$ varies from 0 to 5 with a step of 0.1 ($k>1$ means expansion; $k<1$ means contraction). Our objective is to minimize the average deviation in the reliability plot for forecast of the next one to four weeks. The simulated annealing algorithm proceeds as follows.

1) Starting from a randomly chosen configuration of perturbation intensity, we record the current configuration of perturbation intensity $\boldsymbol{k}_{cnt}$ and the corresponding average deviation in reliability plot $E_{cnt}$.

2) In each iteration $m$, we update $\boldsymbol{k}_{cnt}$ by randomly choosing a predicted lead and adding/subtracting 0.1 from current $k$ value. The updated configuration and average deviation are denoted by $\boldsymbol{k}_{update}$ and $E_{update}$.

3) If $E_{update}<E_{cnt}$, we accept the updated configuration (set $\boldsymbol{k}_{cnt}=\boldsymbol{k}_{update}$ and $E_{cnt}=E_{update}$); otherwise, we accept the updated configuration with probability $\exp(-(E_{update}-E_{cnt})/T)$, where $T={0.9999}^{m}/10$ is set as an exponentially cooling temperature.

4) Iteration stops if $m>{10}^{5}$ or the configuration remains unchanged over 5000 steps.

In Figure I(A), we show the optimized configuration of perturbation intensity at different predicted lead to peak. Inset shows the results from 10 independent optimizations, each starting from different initial conditions. Results have similar patterns for these optimizations. The evolution of average deviation in the reliability plot during the optimization is presented in Figure I(B).

Reliability plots for retrospective forecast

To understand the typical behavior of the forecast method, we display reliability plots for 1- to 4-week prediction, grouped by predicted lead week, i.e., the number of weeks before predicted peak (see Figures J-M). For all 4 targets, the most noticeable deviation of reliability plot from the diagonal line occurs at predicted lead between 0 and 6 weeks, indicating a systematic bias of forecast accuracy for these predictions. With perturbations, this deviation is alleviated, for both training and validation outbreaks. To see this, we show the deviation in the reliability plot for 1- to 4-week prediction in Figure N (training outbreaks) and Figure O (validation outbreaks), with and without perturbation. The distributions of deviation are obtained from bootstrap resampling. In particular, for each predicted lead week and target, we resampled forecasts with replacement for 1,000 times, which produced 1,000 samples of deviation. The boxplots show the distributions of these samples (boxes: 25% to 75% percentiles; whiskers: minimum to maximum). The deviation is consistently reduced after perturbation for all targets. In particular, the most pronounced reduction of deviation is achieved for predicted leads from 0 to 6 weeks.

Log scores for retrospective forecast

We report the distributions of log scores shown in Figure 7 in Table B. In particular, for 1- to 4-week predictions, we report the 5%, 25%, 50%, 75% and 95% percentiles of log score values at each predicted lead to peak. In general, the perturbation procedure dramatically improves the 5% percentiles (bad predictions) at predicted leads between 0 and 6 weeks. This agrees with the improvement observed in Figure 6 (reliability plot) and Figure 7 (log score).

Pseudo codes for optimal perturbation and error correction

The pseudo codes for optimal perturbation and error correction are presented in Algorithm 1 and Algorithm 2. For the optimal perturbation, we compute the fastest error growth direction in the $S-I$ plane using the linearized system, and stretch the ensemble distribution of variables $(S, I)$ along this direction to increase forecast spread. In error correction, we apply the breeding method to diagnose the error structure between two of the most sensitive variables/parameters ($R_{0max}$ and $S$) and the observed variable (incidence). That is, we fit a nonlinear function between errors in $R_{0max}$ or $S$ and errors in the observed variable. With this function, we then estimate errors in $R_{0max}$ and $S$ using the deviation of the prediction from observations. The estimated structural errors in $R_{0max}$ and $S$ are then counteracted for each ensemble member to correct unrealistic trajectories.

| Algorithm 1. Perturbed EAKF |
| --- |
| **Input:** posterior ensemble at week $t$ ${\boldsymbol{\{x}}_{post}^{t}\}$, perturbation intensity $K$. |
| 1. Calculate mean values of variables ($S$, $I$) and parameters ($\beta$, $L$, $D$) using $\{\boldsymbol{x}_{post}^{t}\}$. |
| 2. Set $\eta(S)$ and $\eta(I)$ as the standard deviations of $S$ and $I$ in $\{\boldsymbol{x}_{post}^{t}\}$. |
| 3. Calculate the matrix ${\bar{\boldsymbol{A}}}_{l}+{{\bar{\boldsymbol{A}}}_{l}}^{T}$using Eq. (12) and its principal eigenvector $\boldsymbol{e}_{1}$. |
| 4. Perturb $\{\boldsymbol{x}_{post}^{t}\}$ along $\boldsymbol{e}_{1}$ with intensity $K$. |
| **Output:** the perturbed posterior ensemble $\{\boldsymbol{x}_{post}^{t}\}$. |

| Algorithm 2. Error correction in EAKF |
| --- |
| **Input:** posterior at week $t-1$ $\boldsymbol{x}_{post}^{t-1}$ and week $t$ $\boldsymbol{x}_{post}^{t}$, prior at week $t$ $\boldsymbol{x}_{prior}^{t}$. |
| 1. Diagnosis of error in $R_{0max}$: Add perturbations to $R_{0max}$ from $t-1$ week around the trajectory $\boldsymbol{x}_{bred}^{t-1}=\left( S_{post}^{t-1}, I_{post}^{t-1},obs_{post}^{t-1},{R_{0max}^{t-1}}_{post},{R_{0min}^{t}}_{post},D_{post}^{t},L_{post}^{t} \right)^{T}$, and fit the error structure between observation and $R_{0max}$. Using $\Delta obs=obs_{bred}^{t}-obs_{post}^{t}$, $\Delta R_{0max}$ can be solved from the fitted error structure. The updated $R_{0max}$ at week $t$ is ${R_{0max}^{t}}_{prior}-\Delta R_{0max}$. |
| 2. Diagnosis of error in $S$: Add perturbations to $S$ from $t-1$ week around the trajectory $\boldsymbol{x}_{bred}^{t-1}=\left( S_{post}^{t-1}, I_{post}^{t-1},obs_{post}^{t-1},{R_{0max}^{t}}_{prior}-\Delta R_{0max},{R_{0min}^{t}}_{post},D_{post}^{t},L_{post}^{t} \right)^{T}$, and fit the error structure between observation and $S$. Using $\Delta obs=obs_{bred}^{t}-obs_{post}^{t}$, $\Delta S$ can be solved from the fitted error structure. The updated $S$ at week $t$ is $S_{prior}^{t}-\Delta S$. |
| **Output:** the updated posterior $\boldsymbol{x}_{update}^{t}=\left( S_{prior}^{t}-\Delta S, I_{post}^{t},obs_{post}^{t},{R_{0max}^{t}}_{prior}-\Delta R_{0max},{R_{0min}^{t}}_{post},D_{post}^{t},L_{post}^{t} \right)^{T}$. |

**Table A. Historical outbreaks used for training and validation.** We randomly selected half of all outbreaks for training of perturbation intensity. The outbreaks selected for training are marked with the letter “T”, whereas the remaining outbreaks are marked with the letter “V” and were used for validation.

| City | 03 | 04 | 05 | 06 | 07 | 10 | 11 | 12 | 13 |
| --- | --- | --- | --- | --- | --- | --- | --- | --- | --- |
| BirminghamAL | T | T | V | V | V | T | T | V | V |
| LittleRockAR | V | T | T | V | V | T | V | V | T |
| MesaAZ | V | T | T | T | V | T | T | V | T |
| PhoenixAZ | V | V | V | T | T | V | T | V | T |
| ScottsdaleAZ | T | V | T | V | T | V | V | T | T |
| TempeAZ | V | V | T | V | T | T | T | V | V |
| TucsonAZ | T | T | T | T | T | V | T | V | V |
| BerkeleyCA | V | T | T | T | T | T | T | T | V |
| FresnoCA | V | V | T | T | T | T | V | T | T |
| IrvineCA | V | T | V | V | V | V | T | V | V |
| LosAngelesCA | V | V | V | T | V | V | V | T | V |
| OaklandCA | T | T | V | T | T | V | T | T | V |
| SacramentoCA | V | T | T | T | V | T | T | T | T |
| SanDiegoCA | V | V | V | V | V | V | T | T | V |
| SanFranciscoCA | V | V | V | V | V | T | V | T | V |
| SanJoseCA | V | V | V | V | T | V | T | T | T |
| SantaClaraCA | V | V | V | T | T | V | V | T | T |
| SunnyvaleCA | V | T | T | V | T | V | V | T | T |
| ColoradoSpringsCO | V | T | T | T | V | V | V | T | V |
| DenverCO | V | T | T | V | V | V | T | V | V |
| WashingtonDC | T | V | T | T | V | V | T | T | T |
| GainesvilleFL | T | V | T | V | V | V | T | T | T |
| JacksonvilleFL | T | V | T | V | T | T | T | V | V |
| MiamiFL | T | T | T | T | T | T | T | V | T |
| OrlandoFL | V | V | T | V | V | T | V | V | V |
| TampaFL | V | V | T | V | V | V | V | T | V |
| AtlantaGA | T | T | T | T | V | V | T | V | T |
| RoswellGA | T | T | V | T | V | V | V | T | T |
| DesMoinesIA | T | T | V | T | V | T | T | T | T |
| BoiseID | T | V | V | T | V | V | V | V | T |
| ChicagoIL | T | T | V | T | T | T | T | V | T |
| IndianapolisIN | V | T | T | T | V | T | V | V | T |
| WichitaKS | T | V | T | T | V | V | V | V | V |
| LexingtonKY | T | V | T | V | T | V | T | T | V |
| BatonRougeLA | V | V | V | V | V | T | T | T | V |
| NewOrleansLA | T | V | T | T | V | T | T | T | T |
| BostonMA | T | V | V | V | T | T | V | V | T |
| SomervilleMA | T | T | V | T | T | V | V | V | T |
| BaltimoreMD | T | V | T | T | V | V | T | T | T |
| GrandRapidsMI | T | V | V | T | T | T | V | T | T |
| StPaulMN | T | V | T | T | V | T | T | V | T |
| KansasCityMO | V | T | V | V | T | V | V | V | V |
| SpringfieldMO | T | T | V | V | T | V | T | T | T |
| StLouisMO | T | V | T | T | T | T | T | V | T |
| JacksonMS | T | V | V | T | V | T | T | T | T |
| CaryNC | V | V | T | T | T | T | T | T | V |
| CharlotteNC | T | T | V | T | T | V | T | V | V |
| DurhamNC | V | V | V | T | V | V | V | T | V |
| GreensboroNC | T | T | T | V | T | T | V | V | V |
| RaleighNC | T | V | T | T | T | V | V | T | V |
| LincolnNE | V | V | V | T | T | T | T | V | V |
| OmahaNE | V | T | T | V | V | V | T | T | V |
| NewarkNJ | V | V | T | V | T | V | T | T | T |
| AlbuquerqueNM | T | T | V | T | V | V | V | T | V |
| LasVegasNV | T | T | T | T | V | T | T | T | V |
| RenoNV | T | T | T | V | V | V | T | T | T |
| AlbanyNY | V | T | T | T | V | V | T | T | T |
| BuffaloNY | V | V | T | T | V | V | V | T | T |
| NewYorkNY | T | T | V | T | T | V | V | V | V |
| RochesterNY | T | T | T | T | V | T | V | V | V |
| ClevelandOH | V | T | V | T | T | T | V | T | T |
| ColumbusOH | T | V | T | V | V | T | V | V | V |
| DaytonOH | T | V | V | T | V | T | T | T | V |
| OklahomaCityOK | T | V | T | V | T | V | T | T | V |
| TulsaOK | V | V | T | T | V | T | T | V | V |
| BeavertonOR | V | T | V | V | V | V | T | T | T |
| EugeneOR | T | T | V | T | V | V | V | T | T |
| PortlandOR | V | V | T | V | T | T | V | V | T |
| PhiladelphiaPA | T | T | V | T | V | T | T | V | V |
| PittsburghPA | T | V | V | T | V | T | T | T | T |
| StateCollegePA | T | V | T | V | T | T | T | V | V |
| ProvidenceRI | T | T | T | T | V | V | V | V | V |
| ColumbiaSC | V | T | V | V | V | V | V | T | V |
| GreenvilleSC | V | V | V | T | T | V | T | V | T |
| KnoxvilleTN | T | T | V | T | V | V | T | T | V |
| MemphisTN | T | T | V | T | V | V | V | T | V |
| NashvilleTN | V | T | V | T | T | T | T | V | V |
| AustinTX | T | T | V | T | T | T | T | T | T |
| DallasTX | V | V | T | V | T | V | T | V | V |
| FtWorthTX | T | V | T | T | V | V | T | V | V |
| HoustonTX | V | V | V | T | V | V | V | T | V |
| IrvingTX | T | V | V | V | T | T | V | T | T |
| LubbockTX | T | T | T | V | V | V | V | T | V |
| PlanoTX | V | V | T | V | V | T | T | V | V |
| SanAntonioTX | V | T | V | T | V | T | T | V | T |
| SaltLakeCityUT | V | T | T | V | V | T | T | V | V |
| ArlingtonVA | T | V | T | T | V | T | T | T | V |
| NorfolkVA | V | T | T | T | T | V | T | T | T |
| RestonVA | T | V | T | V | T | T | T | T | T |
| RichmondVA | T | V | V | V | V | V | V | V | T |
| BellevueWA | T | V | V | T | V | V | T | T | V |
| SeattleWA | T | T | V | V | T | V | V | V | T |
| SpokaneWA | T | V | V | T | V | T | V | V | V |
| MadisonWI | V | V | V | T | V | T | T | T | T |
| MilwaukeeWI | V | V | T | T | T | V | V | T | V |

**Table B. Distributions of log scores in Figure 7.** We report the 5%, 25%, 50%, 75% and 95% percentiles of log scores for 1- to 4-week predictions, grouped by predicted lead to peak from -8 weeks to 6 weeks.

| **1-week prediction (5% 25% 50% 75% 95%)** | | | | |
| --- | --- | --- | --- | --- |
| Lead | Train baseline | Train perturbed | Validation baseline | Validation perturbed |
| -8 | -0.02 0.00 0.00 0.00 0.00 | -0.26 -0.09 -0.01 0.00 0.00 | -3.11 0.00 0.00 0.00 0.00 | -2.78 -0.09 -0.02 0.00 0.00 |
| -7 | -0.44 0.00 0.00 0.00 0.00 | -0.90 -0.04 0.00 0.00 0.00 | -0.55 0.00 0.00 0.00 0.00 | -1.11 -0.05 0.00 0.00 0.00 |
| -6 | -1.02 -0.01 0.00 0.00 0.00 | -0.85 -0.14 0.00 0.00 0.00 | -1.17 -0.08 0.00 0.00 0.00 | -1.06 -0.24 0.00 0.00 0.00 |
| -5 | -1.61 -0.11 0.00 0.00 0.00 | -1.14 -0.24 0.00 0.00 0.00 | -2.62 -0.12 0.00 0.00 0.00 | -1.58 -0.29 0.00 0.00 0.00 |
| -4 | -3.00 -0.31 0.00 0.00 0.00 | -2.71 -0.32 0.00 0.00 0.00 | -3.17 -0.19 0.00 0.00 0.00 | -2.76 -0.29 0.00 0.00 0.00 |
| -3 | -4.61 -0.64 0.00 0.00 0.00 | -5.70 -0.84 0.00 0.00 0.00 | -10.00 -0.70 0.00 0.00 0.00 | -5.70 -0.77 0.00 0.00 0.00 |
| -2 | -10.00 -1.63 -0.03 0.00 0.00 | -10.00 -1.94 -0.03 0.00 0.00 | -10.00 -1.77 -0.02 0.00 0.00 | -10.00 -1.73 -0.01 0.00 0.00 |
| -1 | -10.00 -2.41 -0.10 0.00 0.00 | -10.00 -2.13 -0.17 0.00 0.00 | -10.00 -1.93 -0.03 0.00 0.00 | -10.00 -2.04 -0.08 0.00 0.00 |
| 0 | -10.00 -5.70 -1.47 -0.01 0.00 | -8.50 -1.67 -0.68 0.00 0.00 | -10.00 -5.01 -0.94 0.00 0.00 | -6.56 -1.63 -0.55 0.00 0.00 |
| 1 | -10.00 -2.71 -0.45 -0.01 0.00 | -10.00 -2.61 -1.01 -0.04 0.00 | -10.00 -2.53 -0.41 -0.01 0.00 | -10.00 -2.27 -1.07 -0.09 0.00 |
| 2 | -10.00 -1.98 -0.39 -0.04 0.00 | -5.01 -1.41 -0.57 -0.14 0.00 | -10.00 -1.66 -0.34 -0.03 0.00 | -4.09 -1.27 -0.53 -0.09 0.00 |
| 3 | -10.00 -1.36 -0.30 -0.02 0.00 | -2.41 -1.35 -0.77 -0.29 0.00 | -10.00 -1.06 -0.25 -0.03 0.00 | -2.70 -1.34 -0.72 -0.25 0.00 |
| 4 | -5.70 -1.18 -0.08 0.00 0.00 | -2.46 -1.39 -0.45 -0.18 -0.02 | -4.61 -0.81 -0.09 -0.01 0.00 | -2.44 -1.22 -0.45 -0.18 0.00 |
| 5 | -5.01 -0.52 -0.01 0.00 0.00 | -2.41 -1.00 -0.24 -0.08 -0.01 | -4.09 -0.24 -0.01 0.00 0.00 | -2.21 -0.90 -0.23 -0.09 0.00 |
| 6 | -5.70 -0.06 0.00 0.00 0.00 | -2.46 -0.47 -0.11 -0.02 0.00 | -2.91 -0.05 0.00 0.00 0.00 | -1.99 -0.35 -0.09 -0.03 0.00 |
| **2-week prediction (5% 25% 50% 75% 95%)** | | | | |
| Lead | Train baseline | Train perturbed | Validation baseline | Validation perturbed |
| -8 | -3.14 -0.11 0.00 0.00 0.00 | -2.55 -0.44 -0.15 0.00 0.00 | -5.70 -0.13 0.00 0.00 0.00 | -4.35 -0.45 -0.18 -0.01 0.00 |
| -7 | -1.55 -0.21 0.00 0.00 0.00 | -1.79 -0.39 -0.02 0.00 0.00 | -2.15 -0.31 0.00 0.00 0.00 | -2.04 -0.44 -0.03 0.00 0.00 |
| -6 | -2.61 -0.30 0.00 0.00 0.00 | -1.76 -0.52 -0.01 0.00 0.00 | -3.41 -0.46 0.00 0.00 0.00 | -2.17 -0.60 -0.01 0.00 0.00 |
| -5 | -3.06 -0.51 0.00 0.00 0.00 | -2.08 -0.63 0.00 0.00 0.00 | -4.09 -0.68 0.00 0.00 0.00 | -2.48 -0.83 -0.01 0.00 0.00 |
| -4 | -5.70 -0.65 0.00 0.00 0.00 | -5.01 -0.62 0.00 0.00 0.00 | -5.70 -0.71 0.00 0.00 0.00 | -5.36 -0.67 0.00 0.00 0.00 |
| -3 | -10.00 -1.80 -0.01 0.00 0.00 | -10.00 -2.15 -0.01 0.00 0.00 | -10.00 -1.87 0.00 0.00 0.00 | -10.00 -2.09 -0.01 0.00 0.00 |
| -2 | -10.00 -3.16 -0.18 0.00 0.00 | -10.00 -3.62 -0.13 0.00 0.00 | -10.00 -3.31 -0.11 0.00 0.00 | -10.00 -3.51 -0.05 0.00 0.00 |
| -1 | -10.00 -3.40 -0.40 0.00 0.00 | -10.00 -3.91 -0.49 0.00 0.00 | -10.00 -2.68 -0.14 0.00 0.00 | -10.00 -4.09 -0.21 0.00 0.00 |
| 0 | -10.00 -10.00 -1.61 -0.02 0.00 | -10.00 -1.97 -0.82 0.00 0.00 | -10.00 -5.70 -1.40 -0.01 0.00 | -10.00 -1.83 -0.78 0.00 0.00 |
| 1 | -10.00 -3.40 -0.70 -0.04 0.00 | -10.00 -2.57 -1.01 -0.11 0.00 | -10.00 -2.71 -0.61 -0.01 0.00 | -10.00 -2.37 -1.01 -0.10 0.00 |
| 2 | -10.00 -1.95 -0.53 -0.04 0.00 | -5.01 -1.53 -0.60 -0.14 0.00 | -10.00 -1.81 -0.36 -0.03 0.00 | -5.01 -1.36 -0.50 -0.09 0.00 |
| 3 | -10.00 -1.63 -0.17 -0.01 0.00 | -2.66 -1.42 -0.46 -0.21 -0.01 | -10.00 -1.32 -0.19 -0.01 0.00 | -2.79 -1.25 -0.41 -0.19 0.00 |
| 4 | -10.00 -0.77 -0.03 0.00 0.00 | -2.76 -1.15 -0.30 -0.15 -0.02 | -5.01 -0.57 -0.03 0.00 0.00 | -2.65 -1.09 -0.29 -0.15 -0.01 |
| 5 | -10.00 -0.16 0.00 0.00 0.00 | -2.76 -0.97 -0.19 -0.09 -0.02 | -3.93 -0.07 0.00 0.00 0.00 | -2.39 -0.47 -0.17 -0.09 -0.01 |
| 6 | -10.00 -0.02 0.00 0.00 0.00 | -2.44 -0.27 -0.10 -0.03 0.00 | -3.39 -0.01 0.00 0.00 0.00 | -2.17 -0.21 -0.09 -0.04 0.00 |
| **3-week prediction (5% 25% 50% 75% 95%)** | | | | |
| Lead | Train baseline | Train perturbed | Validation baseline | Validation perturbed |
| -8 | -4.39 -0.69 -0.02 0.00 0.00 | -3.80 -1.60 -0.54 -0.02 0.00 | -5.01 -0.79 -0.03 0.00 0.00 | -4.61 -1.54 -0.47 -0.04 0.00 |
| -7 | -3.91 -0.75 -0.01 0.00 0.00 | -3.40 -0.91 -0.07 0.00 0.00 | -3.76 -0.81 -0.01 0.00 0.00 | -3.22 -0.97 -0.12 0.00 0.00 |
| -6 | -4.58 -0.92 -0.01 0.00 0.00 | -3.25 -1.09 -0.04 0.00 0.00 | -5.70 -1.10 -0.03 0.00 0.00 | -3.31 -1.20 -0.06 0.00 0.00 |
| -5 | -5.01 -1.13 -0.01 0.00 0.00 | -3.40 -1.10 -0.03 0.00 0.00 | -5.70 -1.36 -0.01 0.00 0.00 | -4.32 -1.33 -0.05 0.00 0.00 |
| -4 | -10.00 -1.79 -0.01 0.00 0.00 | -10.00 -1.39 -0.02 0.00 0.00 | -10.00 -1.70 -0.01 0.00 0.00 | -10.00 -1.39 -0.02 0.00 0.00 |
| -3 | -10.00 -2.28 -0.06 0.00 0.00 | -10.00 -2.73 -0.04 0.00 0.00 | -10.00 -2.61 -0.06 0.00 0.00 | -10.00 -2.71 -0.03 0.00 0.00 |
| -2 | -10.00 -3.76 -0.48 0.00 0.00 | -10.00 -4.32 -0.46 0.00 0.00 | -10.00 -3.62 -0.35 0.00 0.00 | -10.00 -3.91 -0.19 0.00 0.00 |
| -1 | -10.00 -3.76 -0.60 0.00 0.00 | -10.00 -3.96 -0.57 0.00 0.00 | -10.00 -3.91 -0.38 0.00 0.00 | -10.00 -4.61 -0.42 0.00 0.00 |
| 0 | -10.00 -10.00 -1.88 -0.05 0.00 | -10.00 -2.01 -0.82 0.00 0.00 | -10.00 -5.70 -1.53 -0.03 0.00 | -10.00 -2.12 -0.84 0.00 0.00 |
| 1 | -10.00 -3.14 -0.69 -0.03 0.00 | -10.00 -2.53 -1.09 -0.16 0.00 | -10.00 -2.66 -0.44 -0.01 0.00 | -10.00 -2.48 -0.88 -0.09 0.00 |
| 2 | -10.00 -2.22 -0.33 -0.02 0.00 | -5.70 -1.58 -0.40 -0.06 0.00 | -10.00 -1.50 -0.30 -0.01 0.00 | -4.32 -1.19 -0.39 -0.04 0.00 |
| 3 | -10.00 -1.12 -0.05 0.00 0.00 | -2.96 -0.77 -0.30 -0.15 -0.01 | -10.00 -1.23 -0.05 0.00 0.00 | -2.75 -0.63 -0.28 -0.14 0.00 |
| 4 | -10.00 -0.33 -0.01 0.00 0.00 | -2.93 -0.62 -0.24 -0.13 -0.03 | -5.70 -0.35 0.00 0.00 0.00 | -2.71 -0.52 -0.23 -0.12 -0.01 |
| 5 | -10.00 -0.06 0.00 0.00 0.00 | -2.84 -0.32 -0.15 -0.08 -0.02 | -3.93 -0.02 0.00 0.00 0.00 | -2.42 -0.27 -0.14 -0.08 -0.01 |
| 6 | -5.11 -0.01 0.00 0.00 0.00 | -2.63 -0.20 -0.08 -0.04 0.00 | -3.60 0.00 0.00 0.00 0.00 | -2.48 -0.16 -0.08 -0.04 0.00 |
| **4-week prediction (5% 25% 50% 75% 95%)** | | | | |
| Lead | Train baseline | Train perturbed | Validation baseline | Validation perturbed |
| -8 | -5.70 -1.33 -0.10 0.00 0.00 | -5.01 -2.10 -1.23 -0.06 -0.01 | -5.01 -1.45 -0.13 0.00 0.00 | -5.01 -2.29 -1.11 -0.10 -0.01 |
| -7 | -5.70 -1.48 -0.05 0.00 0.00 | -4.32 -1.58 -0.31 -0.01 0.00 | -5.01 -1.51 -0.08 0.00 0.00 | -3.68 -1.54 -0.36 -0.02 0.00 |
| -6 | -10.00 -1.43 -0.08 0.00 0.00 | -5.01 -1.56 -0.15 0.00 0.00 | -10.00 -1.78 -0.19 0.00 0.00 | -4.61 -1.74 -0.20 0.00 0.00 |
| -5 | -10.00 -1.86 -0.05 0.00 0.00 | -5.01 -1.67 -0.11 0.00 0.00 | -10.00 -1.90 -0.07 0.00 0.00 | -5.70 -1.80 -0.16 0.00 0.00 |
| -4 | -10.00 -2.30 -0.03 0.00 0.00 | -10.00 -1.95 -0.08 0.00 0.00 | -10.00 -2.27 -0.03 0.00 0.00 | -10.00 -2.04 -0.07 0.00 0.00 |
| -3 | -10.00 -3.06 -0.26 0.00 0.00 | -10.00 -3.26 -0.16 0.00 0.00 | -10.00 -3.06 -0.17 0.00 0.00 | -10.00 -3.18 -0.08 0.00 0.00 |
| -2 | -10.00 -3.91 -0.64 0.00 0.00 | -10.00 -4.32 -0.70 0.00 0.00 | -10.00 -3.91 -0.62 0.00 0.00 | -10.00 -3.91 -0.32 0.00 0.00 |
| -1 | -10.00 -4.32 -0.62 0.00 0.00 | -10.00 -4.32 -0.67 0.00 0.00 | -10.00 -3.76 -0.50 0.00 0.00 | -10.00 -5.01 -0.68 0.00 0.00 |
| 0 | -10.00 -5.70 -1.85 -0.08 0.00 | -10.00 -2.28 -0.84 -0.01 0.00 | -10.00 -5.70 -1.47 -0.03 0.00 | -10.00 -1.97 -0.71 0.00 0.00 |
| 1 | -10.00 -3.10 -0.36 -0.01 0.00 | -10.00 -2.76 -0.82 -0.06 0.00 | -10.00 -1.94 -0.27 0.00 0.00 | -10.00 -2.37 -0.70 -0.06 0.00 |
| 2 | -10.00 -1.78 -0.09 0.00 0.00 | -5.70 -1.05 -0.21 -0.02 0.00 | -10.00 -1.21 -0.08 0.00 0.00 | -4.61 -0.77 -0.21 -0.02 0.00 |
| 3 | -10.00 -0.34 -0.01 0.00 0.00 | -3.10 -0.45 -0.23 -0.11 -0.01 | -10.00 -0.44 -0.01 0.00 0.00 | -3.14 -0.37 -0.20 -0.10 0.00 |
| 4 | -10.00 -0.12 0.00 0.00 0.00 | -3.06 -0.34 -0.18 -0.10 -0.02 | -4.32 -0.04 0.00 0.00 0.00 | -2.81 -0.32 -0.17 -0.10 -0.01 |
| 5 | -5.70 -0.02 0.00 0.00 0.00 | -2.85 -0.23 -0.13 -0.07 -0.01 | -3.93 -0.01 0.00 0.00 0.00 | -2.72 -0.20 -0.12 -0.07 -0.01 |
| 6 | -3.35 0.00 0.00 0.00 0.00 | -3.02 -0.16 -0.08 -0.04 0.00 | -2.93 0.00 0.00 0.00 0.00 | -0.88 -0.13 -0.07 -0.04 0.00 |

Figure A. Fitting of historical outbreaks using the SIRS-EAKF system. We fit outbreaks in New York, Denver, Los Angeles and Houston from 2010 to 2013. The red crosses are observed ILI+ signal; blue lines are posterior means; and shaded areas show the 95% CIs of the posterior.

Figure B. Same analysis as in Figure 1 but for perturbations with a standard deviation of $\boldsymbol{\sigma}_{\boldsymbol{p}}\boldsymbol{=5\%}$.

**Figure C. Same analysis as in Figure 1 but for perturbations with a standard deviation of** $\boldsymbol{\sigma}_{\boldsymbol{p}}\boldsymbol{=15\%}$**.**

Figure D. Daily absolute humidity for four cities: New York, Denver, Los Angeles and Houston.

Figure E. Same analysis as in Figure 1 but for the city of Denver.

Figure F. Same analysis as in Figure 1 but for the city of Log Angeles.

Figure G. Same analysis as in Figure 1 but for the city of Houston.

Figure H. Discrepancy between theoretical and simulated error growth rates $\boldsymbol{r(}\boldsymbol{t)}$ for 1,000 synthetic outbreaks. The upper panel shows the distributions of discrepancy between theoretical and simulated values of $\boldsymbol{r(}\boldsymbol{t)}$ at different times. The lower panel presents the distributions of simulated $\boldsymbol{r(}\boldsymbol{t)}$. Compared with the simulated values shown in lower panel, the discrepancy in the upper panel is negligible.

Figure I. Optimization of perturbation intensity using simulated annealing. The optimized perturbation intensity is shown in (A). Inset displays results from 10 independent optimizations, each starting from different initial conditions. The evolution of the average deviation in the reliability plot during the optimization is presented in (B).

**Figure J. Reliability plots for 1-week ahead predictions.** Forecasts are grouped according to predicted lead week, i.e., the number of weeks between the time of forecast initiation and the predicted peak. The x-axis is the average predicted probability $P_{pred}(i)$ and y-axis is the actual observed frequency of occurrence in each category $P_{occur}(i)$. Reliability plots for the forecasts within the group of outbreaks used for training with the baseline forecast approach (train baseline), training with the optimal perturbation approach (train perturbed), validation with the baseline forecasting approach (validation baseline) and validation with the optimal perturbation approach (validation perturbed) are distinguished by different colors.

**Figure K. Reliability plots for 2-week ahead predictions.** Forecasts are grouped according to predicted lead week, i.e., the number of weeks between forecast time and predicted peak. The x-axis is the average predicted probability $P_{pred}(i)$ and y-axis is the actual observed frequency of occurrence in each category $P_{occur}(i)$. Reliability plots for the forecasts within the group of outbreaks used for training with the baseline forecast approach (train baseline), training with the optimal perturbation approach (train perturbed), validation with the baseline forecasting approach (validation baseline) and validation with the optimal perturbation approach (validation perturbed) are distinguished by different colors.

**Figure L. Reliability plots for 3-week ahead predictions.** Forecasts are grouped according to predicted lead week, i.e., the number of weeks between forecast time and predicted peak. The x-axis is the average predicted probability $P_{pred}(i)$ and y-axis is the actual observed frequency of occurrence in each category $P_{occur}(i)$. Reliability plots for the forecasts within the group of outbreaks used for training with the baseline forecast approach (train baseline), training with the optimal perturbation approach (train perturbed), validation with the baseline forecasting approach (validation baseline) and validation with the optimal perturbation approach (validation perturbed) are distinguished by different colors.

**Figure M. Reliability plots for 4-week ahead predictions.** Forecasts are grouped according to predicted lead week, i.e., the number of weeks between forecast time and predicted peak. The x-axis is the average predicted probability $P_{pred}(i)$ and y-axis is the actual observed frequency of occurrence in each category $P_{occur}(i)$. Reliability plots for the forecasts within the group of outbreaks used for training with the baseline forecast approach (train baseline), training with the optimal perturbation approach (train perturbed), validation with the baseline forecasting approach (validation baseline) and validation with the optimal perturbation approach (validation perturbed) are distinguished by different colors.

**Figure N. Reduction of reliability plot deviation achieved by perturbation in retrospective forecast for 1- to 4-week prediction.** Results are averaged over the forecasts of historical outbreaks used for training. The distributions of deviation are obtained from bootstrap resampling.

**Figure O. Reduction of reliability plot deviation achieved by perturbation in retrospective forecast for 1- to 4-week prediction.** Results are averaged over the forecasts of historical outbreaks used for validation. The distributions of deviation are obtained from bootstrap resampling.
